# Exogenous spraying of IAA improved the efficiency of microspore embryogenesis in Wucai (*Brassica campestris* L.) by affecting the balance of endogenous hormones, energy metabolism, and cell wall degradation

**DOI:** 10.1186/s12864-023-09483-2

**Published:** 2023-07-06

**Authors:** Chenggang Wang, Peiyu Zhang, Yun He, Furong Huang, Xu Wang, Hong Li, Lingyun Yuan, Jinfeng Hou, Guohu Chen, Wenjie Wang, Jianqiang Wu, Xiaoyan Tang

**Affiliations:** 1 College of Horticulture, Vegetable Genetics and Breeding Laboratory, Anhui Agricultural University, 130 West Changjiang Road, Hefei, 230036 Anhui China; 2 Provincial Engineering Laboratory for Horticultural Crop Breeding of Anhui, 130 West of Changjiang Road, Hefei, 230036 Anhui China; 3 Wanjiang Vegetable Industrial Technology Institute, Maanshan, 238200 Anhui China

**Keywords:** Wucai, Microspore embryogenesis, IAA, Endogenous hormone, Energy metabolism, Cell wall degradation, ROS

## Abstract

**Background:**

Microspore embryogenesis is an extraordinarily complicated process, comprehensively regulated by a composite network of physiological and molecular factors, among which hormone is one of the most crucial factors. Auxin is required for stress-induced microspore reprogramming, however, the mechanism of its regulation of microspore embryogenesis is still unclear.

**Results:**

In this study, we found exogenously spraying 100 mg·L^− 1^ IAA on the buds of Wucai significantly increased the rate of microspore embryogenesis, and moreover accelerated the process of embryogenesis. Physiological and biochemical tests showed that the contents of amino acids, soluble total sugar, soluble protein, and starch were significantly increased after IAA treatment. Furthermore, exogenously spraying 100 mg·L^− 1^ IAA significantly enhanced IAA, GA_4_, and GA_9_ content, increased catalase (CAT) and malondialdehyde (MDA) activity, and reduced abscisic acid (ABA), MDA and soluble protopectin content, H_2_O_2_ and O_2_·^**−**^ production rate in the bud with the largest population of late-uninucleate-stage microspores. Transcriptome sequencing was performed on buds respectively treated with 100 mg·L^− 1^ IAA and fresh water. A total of 2004 DEGs were identified, of which 79 were involved in micropores development, embryonic development and cell wall formation and modification, most of which were upregulated. KEGG and GO analysis revealed that 9.52% of DEGs were enriched in plant hormone synthesis and signal transduction pathways, pentose and glucuronic acid exchange pathways, and oxidative phosphorylation pathways.

**Conclusions:**

These findings indicated that exogenous IAA altered the contents of endogenous hormone content, total soluble sugar, amino acid, starch, soluble protein, MDA and protopectin, the activities of CAT and peroxidase (POD), and the production rate of H_2_O_2_ and O_2_·^**−**^. Combined with transcriptome analysis, it was found that most genes related to gibberellin (GA) and Auxin (IAA) synthesis and signal transduction, pectin methylase (PME) and polygalacturonase (PGs) genes and genes related to ATP synthesis and electron transport chain were upregulated, and genes related to ABA synthesis and signal transduction were downregulated. These results indicated that exogenous IAA treatment could change the balance of endogenous hormones, accelerate cell wall degradation, promote ATP synthesis and nutrient accumulation, inhibit ROS accumulation, which ultimately promote microspore embryogenesis.

**Supplementary Information:**

The online version contains supplementary material available at 10.1186/s12864-023-09483-2.

## Background

Wucai (*Brassica campestris* L. ssp. *chinensis* var. rosularis Tsen) is a variant of non-heading Chinese cabbage (*Brassica campestris* L.), which is an important species in the Brassicaceae family [1]. As an important autumn and winter vegetable crop, this crop is cultured widely in the Yangtze-Huaihe River Basin. With a high nutritional value, Wucai is becoming an increasingly popular crop in other countries [2]. Breeding homozygous parental lines using traditional plant-breeding methods is difficult because *Brassicaceae* family crops are cross-pollinated. Microspore embryogenesis is the most effective tool of obtaining doubled haploids (DH) plants that are utilized in plant breeding for accelerating production of new cultivars [3].

Microspore embryogenesis involve a complex network of factors [4]. There are a number of intrinsic and extrinsic factors that affect the embryogenesis frequency, including the genotype and physiological status of donor plants, the developmental stage of the microspore, cold pretreatment and heat shock, culture medium (pH, carbohydrates, nitrogen source and other additives) and plant hormones [5]. Although the majority of microspore embryogenesis do not require exogenous hormones as inducers, a transient stress is essential to reprogram of microspores, probably as a response mediated by endogenous hormones.

Among plant growth regulators, auxin is the most significant (*p < 0.05*) hormone in plant development and it is the key regulator of cell division and differentiation [6]. During embryogenesis in planta, the major form of auxin, indoleacetic acid (IAA), has demonstrated key functions, particularly in embryo patterning [7]. The synthesis, activity, and polar transport of indoleacetic acid (IAA) were found to be necessary for the initiation and continuation of microspore embryogenesis in monocotyledonous barley and dicotyledonous *Brassica napus* [8]. Furthermore, gibberellin is also a key regulator of plant growth and development. The two hormones usually work together to control cell division and differentiation. In microspore cultures of *B. napus*, GA_3_ improved plantlet regeneration, mainly via elongation of the embryo axis and acceleration of its maturation [9]. Inhibitor of GA-biosynthesis, uniconazole, applied to *B. napus* embryo at the globular stage of development significantly reduced axis elongation [10]. Besides auxins and gibberellins, abscisic acid (ABA), known as a ubiquitous plant stress hormone, has been claimed to play a role in microspore embryogenesis (ME)-inducing signal transduction system [11]. In recent studies, the function of ABA synthesis and signaling during the in vitro embryogenic process has been analyzed, with *Abscisic Acid Insensitive 3* (*ABI3*) and *ABI4* transcription factors shown to play a relevant role in embryo formation [12]. High frequency of plant development could be obtained by abscisic acid (ABA) treatment of embryos [13]. On the contrary, higher level of ABA significantly inhibited embryogenesis.

Plant cell wall is complex and dynamic structures that involved in a series of functions such as interaction with the environment, intercellular communication, regulation of plant growth and development, and the determination of cell size and shape [14]. Previous studies indicated that cell wall remodeling plays an indispensable role in microspore embryogenesis [15]. Pectin are the family of oligosaccharides and polysaccharides with common characteristics and the major component of the primary wall [16], which play a vital role in microspore embrogenesis [15]. Pectin exist in the cell wall with highly methylated form, and its methylation level is mainly regulated by pectin methyl esterases (PMEs) [17]. PMEs catalyze the removal of methyl esters from homogalacturonan backbone of pectins in plant cell wall. The de-methylesterified residues can either form Ca^2+^ bonds or turn into a target for polygalacturonases, one of pectin-degrading enzymes, which affect the texture and rigidity of the cell wall [18]. In *Brassica napus*, Changes in pectin esterification levels are relevant to temporal expression patterns of *PME* genes in microspore embryogenesis [14]. De-methylesterified pectins are enriched in cell and *PME* genes are highly expressed in torpedo and cotyledon embryos of *Brassica napus*. Furthermore, inhibition of PME activity led to impair somatic embryogenesis in *Quercus suber*[19], indicating that pectins esterification play a role in inducing embryogenesis. Testillano et al. considered that PME-mediated configuration variation of pectins could be a crucial factor for microspore mebryogenesis, acting by promoting the cell wall remodeling in the process [15].

Intracellular reactive oxygen species (ROS) levels have different degrees of influence on microspore development. In previous studies, ROS were involved in the programmed cell death (PCD) of tapetal cells [20, 21]. The abnormal metabolism of ROS during plant PCD leads to delayed tapetal degradation, insufficient nutrition supply for microspore development, and microspore abortion [22–24]. To maintain the balance of intracellular ROS, plants have evolved a series of clear mechanisms for enzymatic and non-enzymatic ROS [25]. The activity of antioxidant enzymes plays an important role in clarifying free radicals and regulating cell tissue activity during embryogenesis [26].

In this study, the optimal concentration of exogenous IAA was identified in Wucai, which significantly (*p < 0.05*) increased the rate of microspore embryogenesis and moreover accelerated the process of embryogenesis. RNA-seq analysis suggested that plant hormone synthesis and signal transduction pathways, pentose and glucuronic acid exchange pathways, and oxidative phosphorylation pathways were involved in the regulation of microspores embryogenesis. Combined with analysis of changes in endogenous hormones and physiological indicators and transcriptome analysis, it was found that exogenous IAA treatment could change the balance of endogenous hormones, accelerate cell wall degradation, promote ATP synthesis and nutrient accumulation, inhibit ROS accumulation, which ultimately promote microspore embryogenesis.

## Results

### Effects of IAA on the rate of microspore embryogenesis

**Fig. 1:**
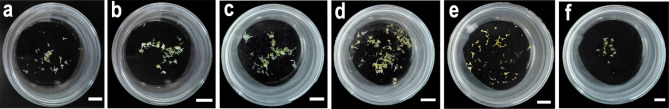
Images showing the embryogenic potential of isolated microspores of Wucai treated with different concentrations of IAA: **(a)** 0 mg·L^− 1^ IAA; **(b)** 50 mg·L^− 1^ IAA; **(c)** 75 mg·L^− 1^ IAA; **(d)** 100 mg·L^− 1^ IAA; **(e)** 125 mg·L^− 1^ IAA; and **(f)** 150 mg·L^− 1^ IAA. Bar = 1 cm

The rate of isolated microspore-derive embryogenesis in Wucai was significantly (*p < 0.05*) increased treated with IAA (Fig. 1). Statistical analysis showed that IAA treatment enhanced the occurrence of embryoid bodies in a dose dependent manner (Table 1). Low concentration of exogenous IAA had little or no effect on the incidence of microspore embryogenesis, while high concentration even inhibited microspore embryogenesis. This experiment found that 100 mg. L ^− 1^ IAA treatment had the best effect on induction of embryogenesis, the embryo yield reached 16.4 embryos. bud ^− 1^, 3.15 times higher than that in the control group. At the same time, 150 mg. L ^− 1^ IAA inhibited the development of isolated microspore embryos. These results indicate that treatment with an appropriate IAA concentration is conducive to the embryogenesis of isolated microspores in Wucai.

### Cytological observation of microspore embryonic development

According to the first division of microspore, the embryonic development pathways were divided into two types: Route A (unequal division) and Route B (equal division). Route A can be further subdivided according to the second division and subsequent situations. Studies had shown that there were two pathways, A and B, for embryonic development in isolated microspore cultures of non-heading Chinese cabbage, and pathway A was more common [27].

**Fig. 2:**
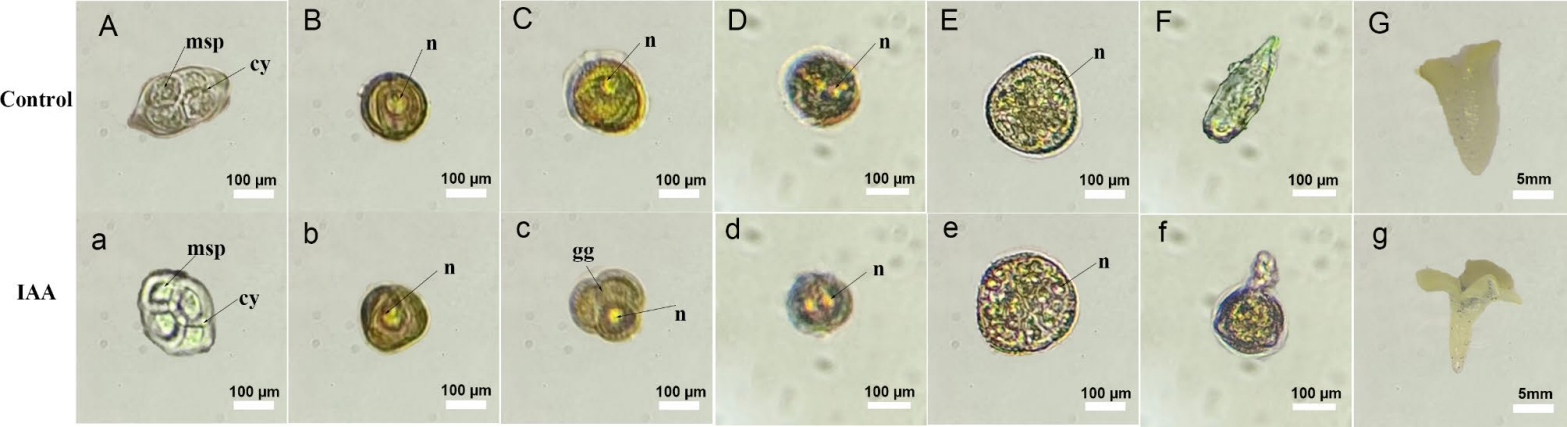
Cytological observation of isolated microspore development between the control (A–F) and IAA-treated (a–f) groups. **A(a)** tetrad stage (0 d). **B(b)** early uni-nucleated microspore stage (0 d). **C(c)** late uni-nucleated microspore stage (0 d). **D(d)** bi-nucleated microspore stage (3 d). **E(e)** Multicellular proembryo (5 d). **F(f)** Embryoid structure (10 d). **G(g)** Embryoid (15 d). msp, microspore, cy, cytoplasm, n, nucleus

In this study, the microspore mother cells underwent meiosis to form a tetrad (Fig. 2), and four parenchyma cells were surrounded by a thick layer of callose (Fig. 2A, a). The tetrad was separated to form isolated microspores. The microspore just separated from the tetrad was in the mononuclear metaphase, when the nuclear to cytoplasm ratio was large (Fig. 2B, b). Subsequently, the nucleus was pushed aside by vacuoles, i.e., the late uni-nucleated stage (Fig. 2C, c), the most sensitive stage for the induction of microspore embryogenesis. The microspore undergoes mitosis and forms two cell nucleus, one large and one small (Fig. 2D, d), i.e. unequal division. Therefore, we speculated that the developmental pathway of isolated microspores in Wucai was pathway A.

Compared to the control, the IAA treated microspores had a dispersed arrangement of four microspores during the tetrad stage (Fig. 2b). At the mid-mononucleate stage, the nucleus was significantly larger, and at the mononucleate leaning stage, it was triclinic with a distinct budding furrow, in preparation for subsequent divisions (Fig. 2c). The late uni-nucleate stage (Fig. 2d) was the spmpling stage of the buds in our experiment, as it was the best stage for inducing microspore embryogenesis [28, 29]. In the 3rd day of isolated microspore culture, the microspores were observed to undergo unequal division, forming a large vegetative nucleus and a small reproductive nucleus (Fig. 2d). On the 5th day of culture, the multicellular clusters formed were regular and round. (Fig. 2e). On the 10th day, in the control group, the early embryos were mostly torpedo shaped (Fig. 2F, G), while in the IAA treated group, the early embryos first formed a long stalk and eventually developed into cotyledons (Fig. 2f, g). The multicellular proto embryos divided further in culture and eventually formed microspore embryos. In addition, the related research indicated that the microspore embryo would pass through the globular embryo, the heart shaped embryo, the torpedo shaped embryo and finally develop into the cotyledonary embryo [28]. In this study, microscopic observation of microspores after 5 days of culture and statistical analysis of 10 randomly selected fields of view showed that the number of multicellular proembryos treated with IAA was significantly higher than that of the control group (fresh water treatment) (Additional file 1: Fig. s1) The initial time of microspore embryogenesis was counted after spraying with different concentrations of IAA (Additional file 2: Table s1). Compared with the control group, the time of microspore embryogenesis was 4 d earlier after 100 mg. L^− 1^ IAA treatment.

### Comparison of changes in the content of endogenous plant hormones

**Fig. 3:**
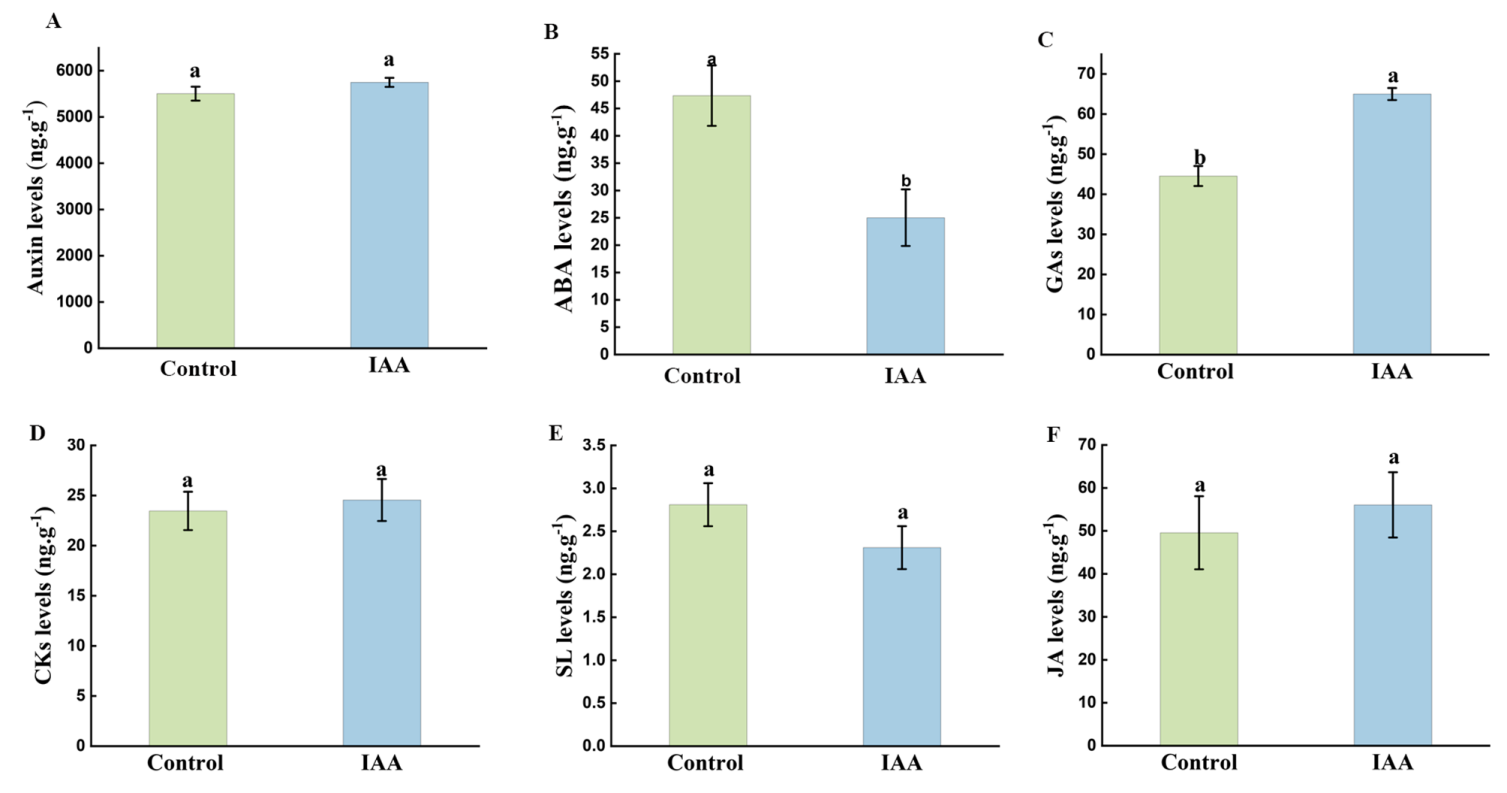
Determination of endogenous plant hormone content between buds of the control a nd IAA treatment groups. **(A)** Auxin content, **(B)** cytokinin content, **(C)** gibberellin content, **(D)** abscisic acid content, **(E)** jasmonic acid content, and **(F)** monolayer content. Data were derived from three independent experiments. Error bars represent ± SD. Different letters indicate significant differences (*p* < 0.05)

After exogenous IAA treatment, 0.3 g of fresh flower bud samples were used to determine the endogenous levels of plant hormones such as auxin, CKs, ABA, GAs, JA and SLs. Compared to the control, the content of GAs in monocotyledonous leaning stage flower buds was dramatically increased, and the ABA content was significantly (*p < 0.05*) decreased after exogenous IAA treatment, while the content of other phytohormones did not show obvious (*p < 0.05*) changes (Fig. 3).

In the plant system, the physiological effects of different categories of plant hormones have a mutually reinforcing or antagonistic effect, and the regulation of plant growth and development is usually the result of the combined action of multiple hormones. Therefore, the ratio of several hormones was analyzed in this experiment. The ratios of (auxin + GAs)/ABA, (auxin + GAs + CKs)/ABA, GAs/ABA, and auxin/ABA were all higher in the treatment group than in the control group (Addition File 3: Table s2), while the ratios of (auxin + GAs)/CKs and auxin/CKs were not obviously different from those in the control group. These results indicate that microspore development may also be related to the relative content and the dynamic balance between hormones.

### Transcriptome sequencing data and quality control

To explore the molecular mechanism of exogenous IAA in enhancing the embryogenesis of isolated microspores in Wucai, we performed transcriptomic analysis using the buds of the largest population of late-uninucleate-stage were analyzed after treatment with 100 mg. L^− 1^ IAA and the same dose of water for 24 h. After sequencing quality control and data analysis, 256,144,226 clean reads were obtained, and the clean data of each sample reached more than 5 GB. The percentage of Q30 was more than 92% (Table 2). In a direct comparison of the density and discrete distribution of expression levels for different samples, the sequencing quality and gene expression levels were the same (Additional file 4: Fig s2). These indicated that the sequencing quality was good, and could be analyzed in the next step.

**Table 1:** Effect of exogenous IAA on embryo rate of isolated microspores

IAA Concentration (mg. L^− 1^)	Frequency of microspore-derived embryo (No.of embryos per bud, Mean ± SD)
0	5.20 ± 0.53d
50	6.10 ± 0.12d
75	11.10 ± 0.28c
100	16.40 ± 0.53a
125	13.48 ± 0.23b
150	4.76 ± 0.13e

Note: In *t* test, different small letters in the same column of data indicated significant differences (*P* < 0.05)

**Table 2:** Illumina sequencing data and results of de novo assembly

Sample	Total Reads	Clean Base(G)	Q30(%)	GC-content (%)	Reads- mapped	Unique-mapped	Multi-mapped
Control-1	56,593,044	8.49	94.1	46.41	49,432,181 (87.35%)	85.40%	1.95%
Control-2	38,553,118	5.78	92.1	46.24	33,444,692 (86.75%)	84.84%	1.91%
Control-3	39,350,566	5.9	92.35	46.55	34,297,010 (87.16%)	85.19%	1.97%
IAA-1	39,273,240	5.89	92.35	46.16	34,309,235 (87.36%)	85.22%	2.14%
IAA-2	39,439,958	5.92	92.57	46.19	34,243,135 (86.82%)	84.71%	2.11%
IAA-3	42,934,300	6.44	92.39	46.59	37,536,097 (87.43%)	85.46%	1.97%

### Identification of differentially expressed genes

To better understand the expression patterns of differentially expressed genes (DEGs), DESeq was used for differential expression analysis [30, 31]. DEGs between the buds treated with 100 mg. L^− 1^ IAA and control group were identified. The screening conditions of differential genes were as follows: absolute value of log_2_(FoldChang) greater than or equal to 1, and the p-value less than 0.05. A total of 2004 DEGs were identified, comprising 1002 upregulated genes and 1002 downregulated genes. In addition, genes that were not compared to the reference genome were considered new, and we identified 345 new genes, 52 of which could be not annotated to any database.

### GO and KEGG analysis of differentially expressed genes

**Fig. 4:**
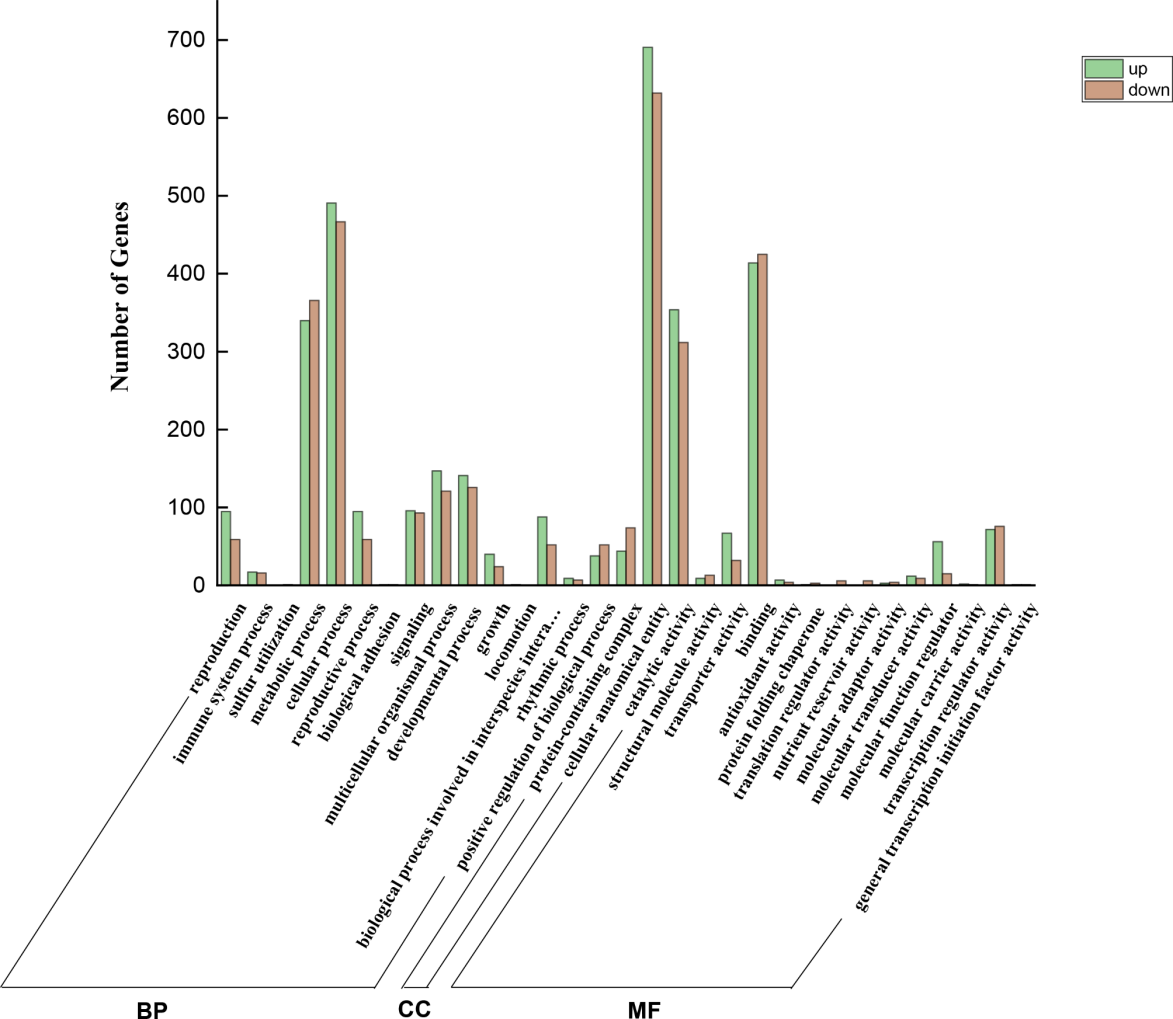
GO enrichment of flower bud DEGs between the control and IAA-treated groups

Detailed GO term annotations of the DEGs were categorized according to biological processes, molecular functions, and cellular components. The GO categories for the set of DEGs (Fig. 4) revealed that most of the encoded products were associated with “metabolic process”, “cellular process”, “multicellular BPs”, “developmental process”, “cellular anatomical entity”, “catalytic activity” and “binding”.

**Fig. 5:**
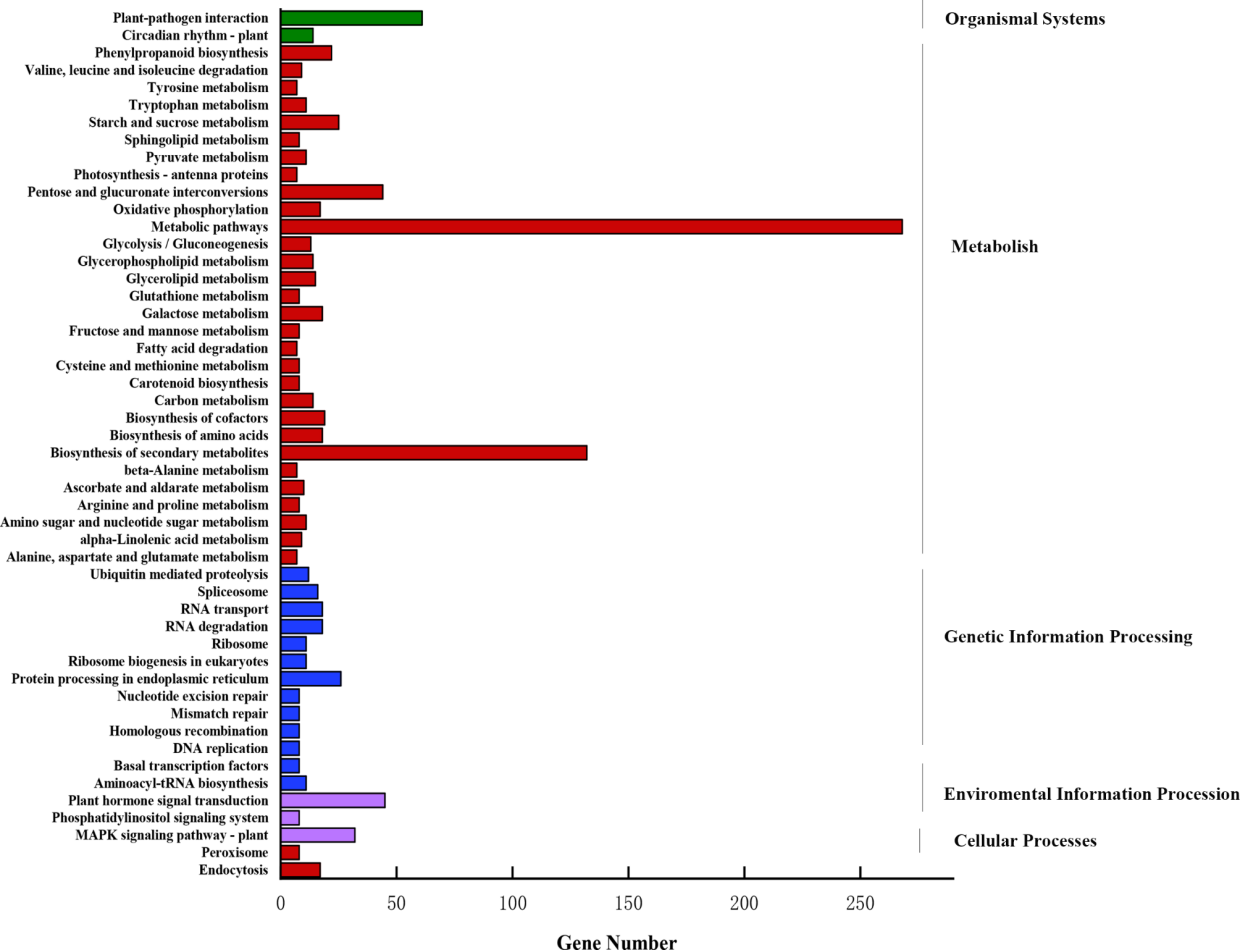
First 50 pathway entries for KEGG enrichment of bud differentially expressed genes (DEGs) between the control and IAA-treated groups

KEGG [32] ( www.kegg.jp/kegg/kegg1.html ) enrichment analysis showed that 1334 differential genes were enriched in 121 metabolic pathways (Additional file5, Fig s3). The metabolic pathways were annotated with “metabolic pathway”, “pentose and glucuronate interconversions”, “plant-pathogen interaction”, “biosynthesis of secondary metabolism”, “plant hormone signal transduction” and “oxidative phosphorylation” (Fig. 5). These results indicated that a variety of complex metabolic pathways were involved in microspore development.

### Pollen and embryo development related genes

Pollen development was a complex process involved many events, including microspore embryonic development. Many genes related to pollen and embryonic development have been identified in the model plant *Arabidopsis thaliana* [33, 34]. A total of 79 DEGs were identified as being associated with pollen and embryonic development, which 46 were upregulated and 23 were downregulated (Additional file 6; Table s3), including GDSL lipase genes, BHLH transcription factor family genes, AP2/ERF transcription factor family genes, pectinesterase genes, WUS-related genes, GST (glutathione S-transferase) related genes, and RALF (protein RALF-like) genes. These results indicate that these DEGs participate in pollen and embryonic development, as well as reproductive development, and play a certain role in promoting the subsequent embryogenesis of isolated microspores.

Based on GO and KEGG enrichment analysis and identification of development-related genes, we screened out 139 genes under the condition of |log_2_FC|>=2, *P* < 0.05, and presented them in the heatmap (Additional file 7: Fig s4).

### Transcriptional and metabolic regulatory networks for plant hormone synthesis and signal transduction

**Fig. 6:**
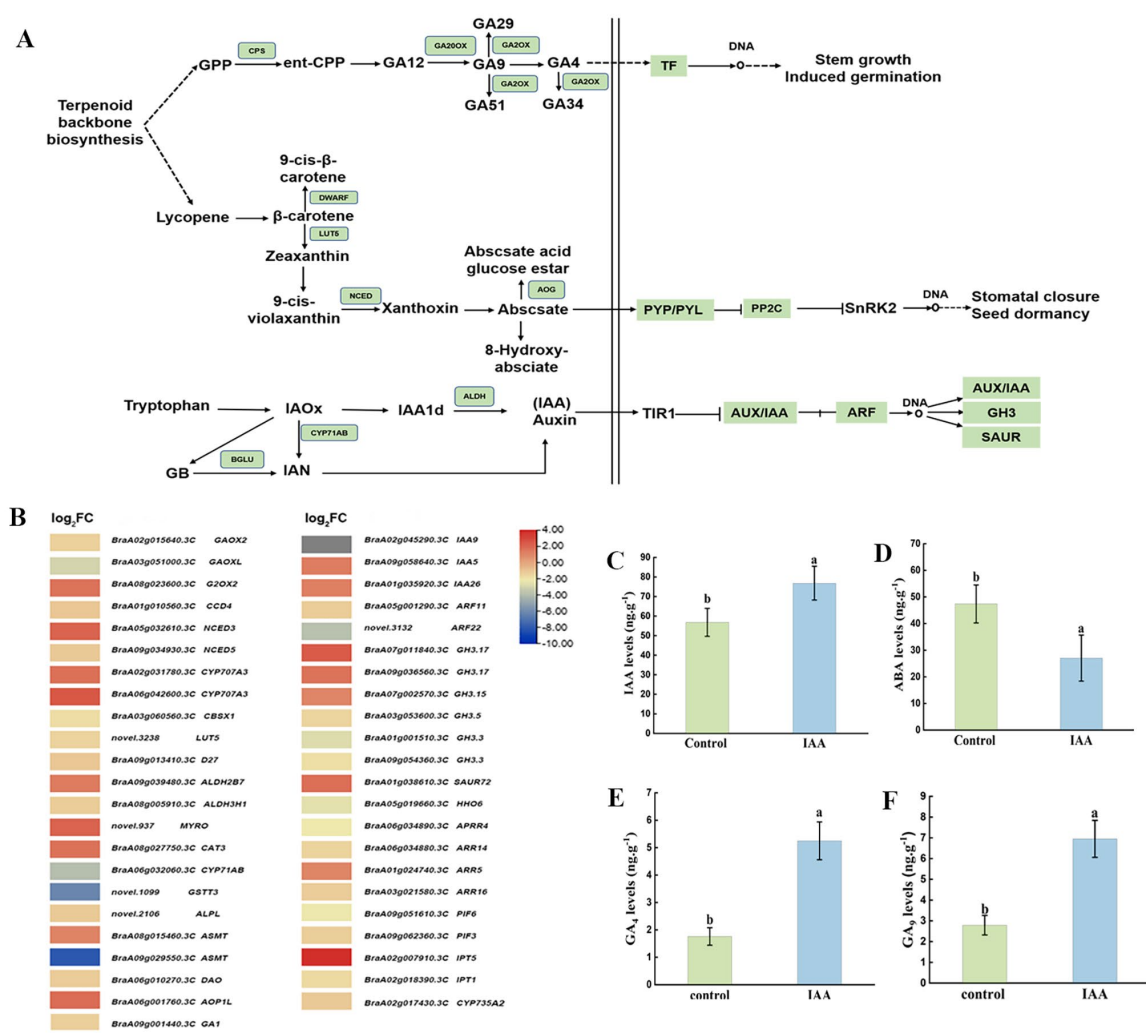
Metabolic pathway of plant hormone synthesis and signal transduction between the control and IAA-treated groups. **(A)** Plant hormone synthesis and signal transduction metabolic pathway (ko04075, ko00904, ko00308, ko00906). **(B)** Heat map of differentially expressed genes (DEGs) related to plant hormone synthesis and signal transduction. **(C)** IAA content. **(D)** ABA content. **(E)** GA_4_ content. **(F)** GA_9_ content. Data were derived from three independent experiments. Error bars represent ± SD. Different letters indicate significant differences (*p* < 0.05)

To explore the role of the regulatory network of genes and metabolites in plant hormone synthesis and signal transduction, all detected DEGs and metabolites were mapped to the related pathways (Fig. 6A, B).

In the process of gibberellin synthesis (ko00904), non-bioactive GA_24_ was converted into active GA_4_ and GA_9_ under the catalysis of the *GA20OX* enzyme. *GA2OX*, as a key enzyme in the degradation process of GAs in plants, oxidizes bioactive GA*4* to inactive GA_34_, and oxidizes GA_9_, the precursor of GA_4_, to inactive GA_51_ and GA_29_, thereby maintaining the dynamic balance between bioactive GAs and their intermediates in plants. During phytohormone signaling (ko04075), the transcription factors *PIF3* and *PIF4* were activated to mediate the gibberellin signal response. GA_4_ (Fig. 6E) and GA_9_ (Fig. 6F) contents were remarkably higher than that of the control group.

During auxin biosynthesis (ko00380), tryptophan is catalyzed by enzymes to synthesize indole 3 acetamide (IAM) and indole 3 acetaldehyde (IAAld), prerequisite substances of IAA. IAA1d is synthesized into indoleacetic acid through the action of aldehyde dehydrogenase. In our study, *CYP71AB* was downregulated, and *DAO* was upregulated. There were four genes encoding aldehyde dehydrogenase (*ALDH*), of which one was upregulated and three were downregulated. During auxin signal transduction (ko04075), more than 60% of DEGs were upregulated. At the same time, we found that the content of IAA was significantly (*p < 0.05*) higher than that in the control group (Fig. 6C). Changes in these DEGs may have contributed to the accumulation of IAA.

During ABA synthesis (ko00906), β-cyclohydroxylase (*LUT5*), β-carotene isomerase (*DWARF27*), and abscisic β-glucosyltransferase (*AOG*) were all downregulated, while abscisic acid 8’-hydroxylase (*CYP707A*) was upregulated. Leading to a decrease in ABA accumulation in vivo. Both the abscisic acid receptor (*PYL*) and protein phosphatase (*PP2C*) were downregulated during signal transduction (ko04075). This was consistent with our detection of a significant reduction (*p < 0.05*) in ABA content (Fig. 6D).

### Oxidative phosphorylation pathway

**Fig. 7:**
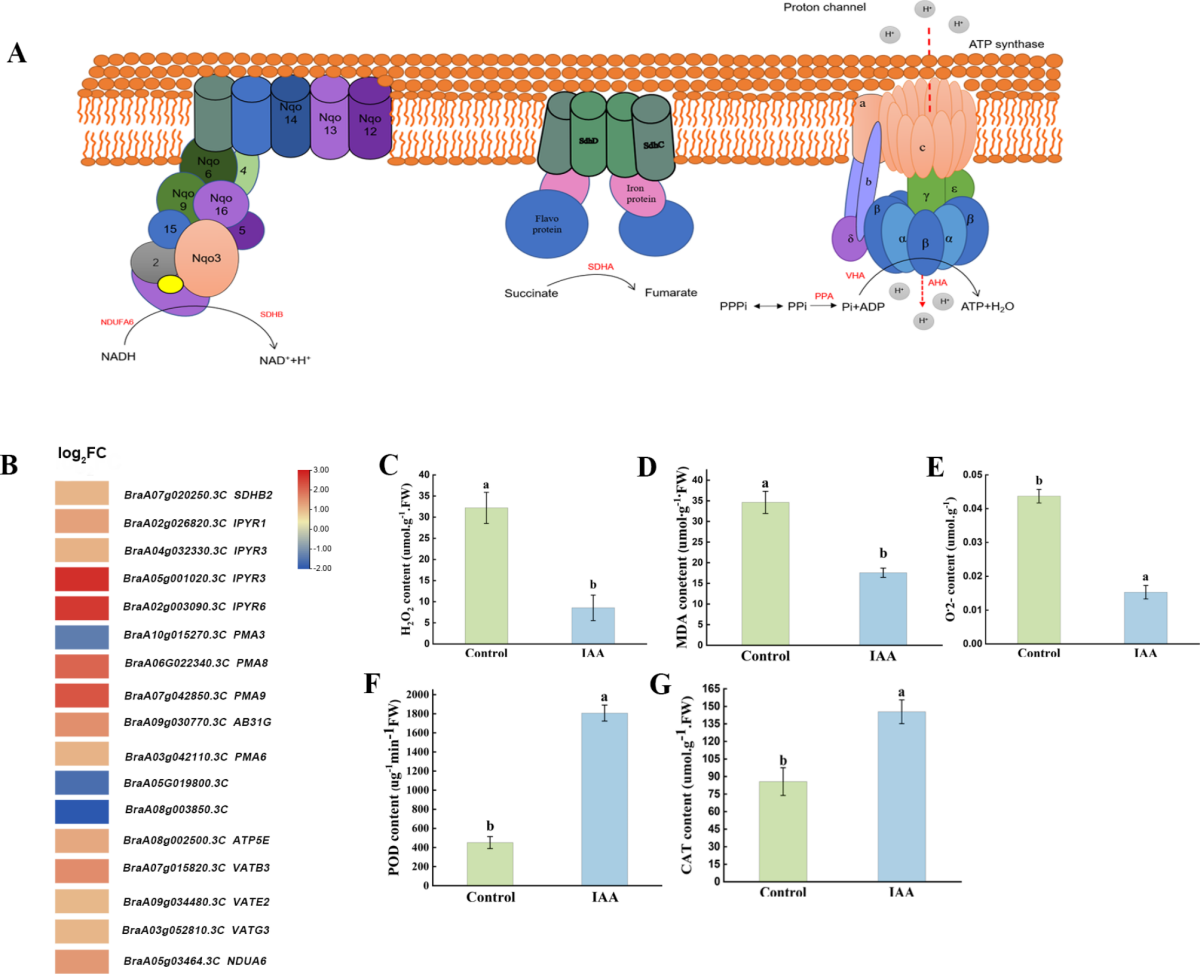
Metabolic pathways of oxidative phosphorylation between the control and IAA-treated groups. **(A)** Oxidative phosphorylation metabolic pathway (ko00190). **(B)** Heat map of oxidative phosphorylation metabolism-related differentially expressed genes (DEGs). **(C)** H_2_O_2_ content. **(D)** MDA content. **(E)** O2.^−^ content. **(F)** POD content. **(G)** CAT content. Data were derived from three independent experiments. Error bars represent ± SD. Different letters indicate significant differences (*p* < 0.05)

Normal microspore development requires an adequate energy supply, and the energy produced by mitochondrial oxidative phosphorylation (ko00190) in plants supplies the needs for plant growth and breeding. We plotted a model of oxidative phosphorylatipn metabolism (Fig. 7A). A total of 17 DEGs were detected in the oxidative phosphorylation pathway, of which 14 were upregulated and 3 were downregulated (Fig. 7B). DEGs were enriched in NADH dehydrogenase, succinate dehydrogenase and ATP synthase (complex V), which were involved in electron transfer and ATP synthesis. Exogenous IAA treatment significantly (*p < 0.05*) decreased H_2_O_2_ (Fig. 7C), MDA (Fig. 7D), and O_2_· ^**−**^ (Fig. 7E) contents, and enhanced POD (Fig. 7F), CAT (Fig. 7G) activity, which maintained the balance of ROS levels during microspore development and prevented ROS accumulation, reducing membrane damage.

**Fig. 8:**
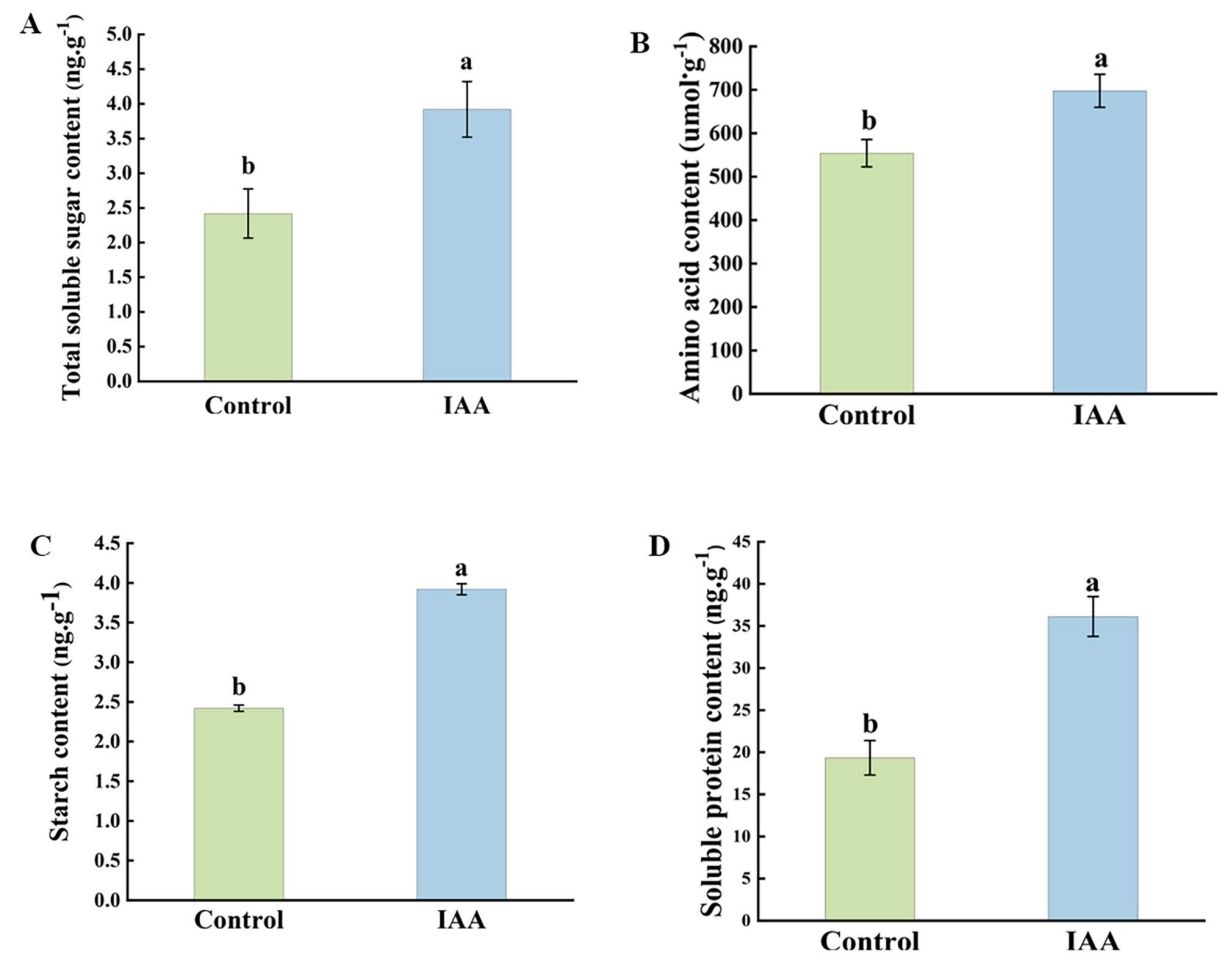
Determination of physiological indices. **(A)** Total soluble sugar content, **(B)** glucose content, **(C)** starch content, and **(D)** soluble protein content. Data were derived from three independent experiments. Error bars represent ± SD. Different letters indicate significant differences (*p* < 0.05)

To further prove that the development of microspores was related to the energy supply, the energy substances and nutrient substances were measured. Compared with the control group, the total soluble sugar (Fig. 8A), amino acid (Fig. 8B), starch (Fig. 8C) and soluble protein content (Fig. 8D) in buds dramatically increased (*p < 0.05*) after treated with IAA.

### Pentose and glucuronate interconversions pathway

**Fig. 9:**
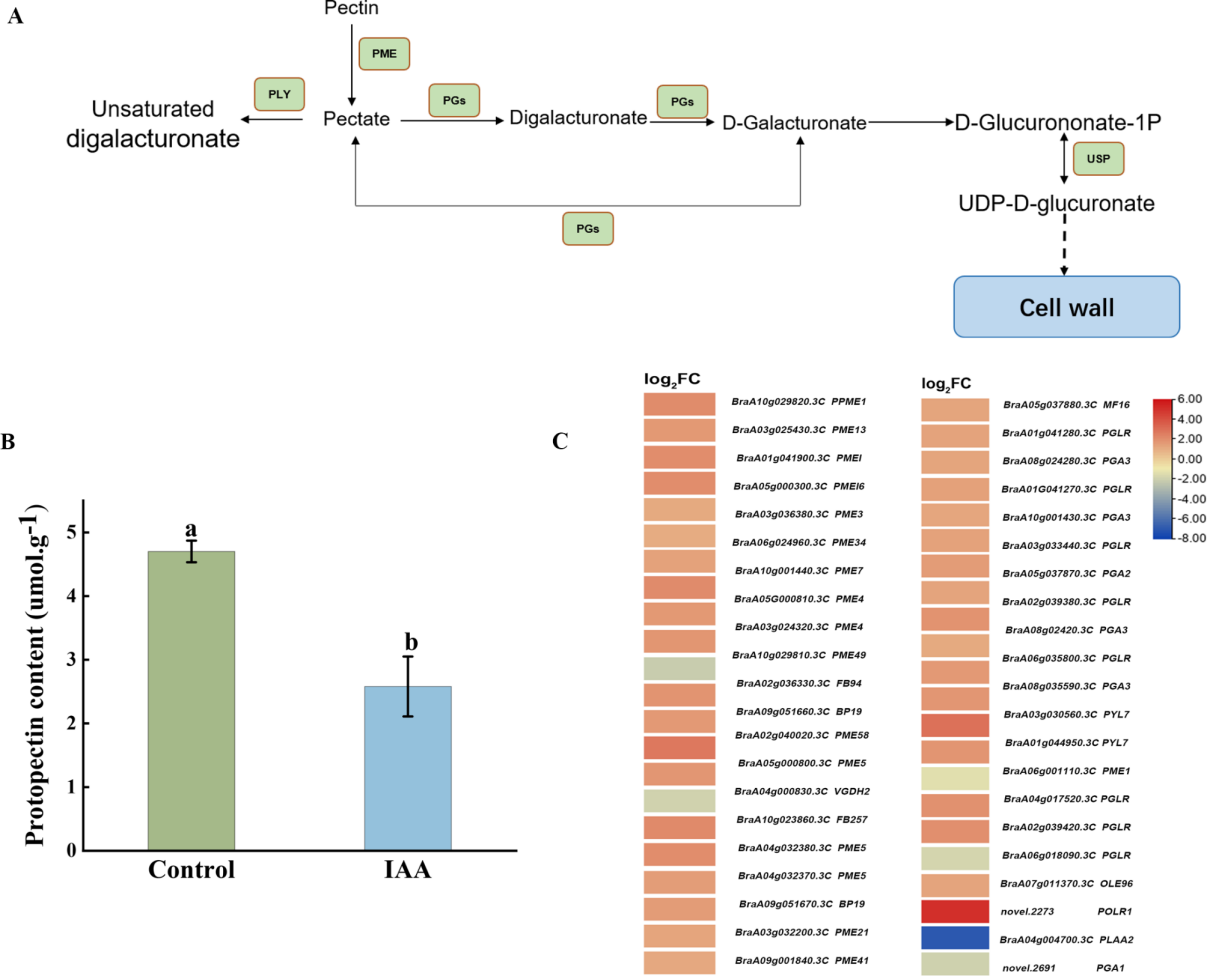
Pentose and glucuronide exchange pathways between the control and IAA-treated groups. **(A)** Pentose and glucose exchange pathway (ko00040). **(B)** Heat map of the differentially expressed genes (DEGs) associated with the pentose and glucuronic acid interchange pathway. **(C)** Pectin content. Data were derived from three independent experiments. Error bars represent ± SD. Different letters indicate significant differences (*p* < 0.05)

According to the KEGG analyses, the pentose and glucuronic acid exchange pathway (ko00040) was significantly enriched. The regulatory pattern of related differential genes in the metabolic pathway was shown in Fig. 9A. In the pentose and glucuronate interconversion pathway, more than 80% upregulated expression of DEGs were detected, including the pectin methylase (*PME*) and polygalacturonase (*PGs*), key enzymes in pectin metabolism, encoding polygalacturonases (*GSVIVT*) and UDP-sugar pyrophosphorylase (*USP*) (Fig. 9C). The pectins content were significantly (*p < 0.05*) lower than that of the control (Fig. 9B).

### The expression patterns of DEGs are verified by RT-qPCR

**Fig. 10:**
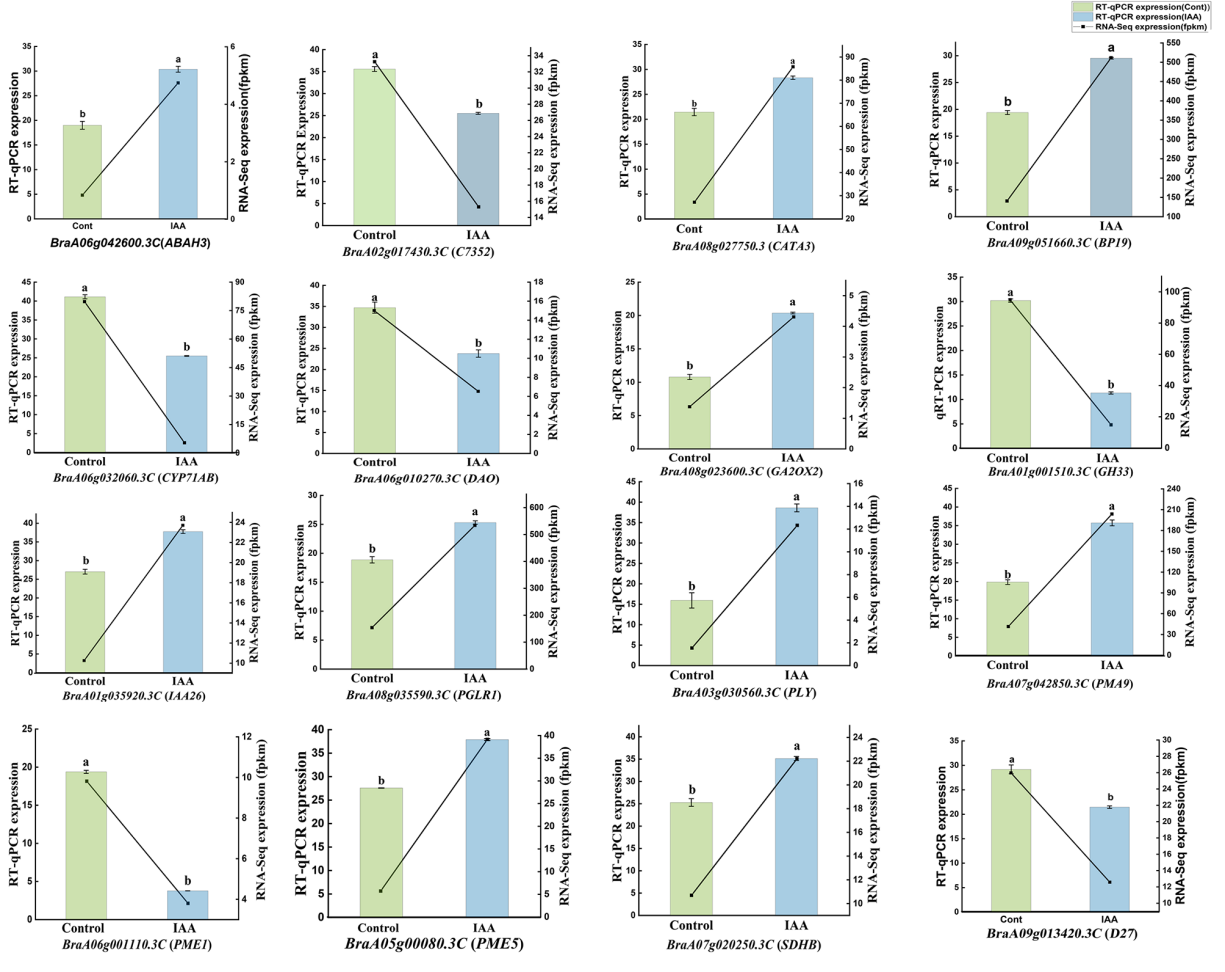
Expression levels of DEGs according to RT-qPCR (histogram) and RNA-Seq (black line chart). The FPKM values are based on the RNA-Seq data. The data obtained by RT-qPCR represent the means based on three replicates

16 DEGs were selected from the important metabolic pathways for RT-qPCR to verify the reliability of RNA-Seq (Fig. 10). These DEGs were involved in plant hormone synthesis and signal transduction (*IAA26*, *D27*, *DAO*, *GA2OX2*, *CYP71A13*, *ABAH3*, *C7352*, *GH33*, *CED3*), pentose and glucuronic acid exchange pathways (*PME5*, *PME1*, *PGLR1*, *PLY*, *BP19*), oxidative phosphorylation metabolic pathways (*SDHB*, *SDB19*). The gene expression patterns obtained from RT-qPCR and RNA-Seq data showed similar trends, which confirmed the accuracy of the RNA-Seq results obtained in this study.

## Discussion

### Treatment with exogenous IAA to initiate microspore embryogenesis by affecting the balance of endogenous hormones

Hormones are an important factor affecting the development of microspore embryos. Auxin biosynthesis, transport, and response severely affect embryo development [35] IAA can be synthesized via the IPA pathway, the IAOx pathway, and other pathways [36]. The IAOx pathway may be a species-specific pathway for *Brassicaceae* species [37–39]. Premature degeneration of the tapetum during microspore development leads to cytoplasmic male sterility, and the *ALDH* gene has the function of overcoming this phenomenon [40]. The primary function of *CYP71A13* is to catalyze the conversion of IAOx to IAN [41]. The regulation of active plant hormone levels is possible at β-glucosidases (*BGLU*) [42]. In this study, *CYP71A13* was downregulated, and *BGLU* was upregulated; one gene encoding *ALDH* was upregulated. During plant hormone signal transduction, *SAUR*, and *AUX/IAA*, which are annotated as auxin-responsive proteins, are upregulated with high signal transduction levels. Related research shows that *AUX* prevents auxin leakage by transporting extracellular auxin back to the cytoplasm [43]. This may be the reason why the endogenous IAA content is significantly higher than that of the control group.

Studies have shown that exogenous IAA applications can improve endogenous GA biosynthesis [44]. The production of embryonic cells increased as GA content increased, and the amount of bioactive GA positively correlated with the formation of somatic embryos [45]. After treatment with exogenous 100 mg. L^− 1^ IAA, the contents of endogenous GAs, GA_4_and GA_9_ were significantly increased. In the GA biosynthesis process, the expression of *GA*_*20OX*_ and *GA*_*2OX*_ regulates the level of GA with biological activity in vivo. The molecular mechanism of GA on microspore embryogenesis needs to be further studied.

ABA accumulation inhibits the occurrence of microspore embryos, and low concentration of ABA promotes the occurrence of microspore embryos [46]. The absence of *LUT5* results in a decrease in beta-carotene-derived lutein [47]. *DWARF27* is a protein that converts trans-carotene to 9-cis-carotene [48]. 9-Cis-epoxycarotenoid dioxygenase (*NCED*) plays a key role in the development of ABA synthesis in plants [49]. Abscisic acid 8’-hydroxyase (*CYP707A*) is a key enzyme for ABA catabolism [50]. ABA seems to play a predominant role in the conversion of environmental signals into changes in the gene expression of plants. In this study, *DWARF27*, *LUT5*, *NCED*, and abscisic beta-glucosyltransferase (*AOG*) were downregulated, while *CYP707A* was upregulated. Both abscisic acid receptors (*PYR/PYL*) and protein phosphatase 2 C *(PP2C*) were downregulated, i.e., so ABA synthesis and transcription levels were low. This was consistent with significant decrease in ABA content.

The balanced relationship between plant hormones affects microspore development and embryogenesis [51]. In this study, the ratio of endogenous hormone contents in flower buds at the uni-nuclear stage after treatment with 100 mg. L^− 1^ IAA was statistically analyzed (Additional file 3: Table s2), and changes in hormone balance were found, which were consistent with the results of previous studies [51, 52]. Based on the analysis of plant hormone synthesis and signal transduction pathways, as well as the results of endogenous plant hormone content determination, we speculated that exogenous IAA affected the occurrence of isolated microspore embryos by affecting the level of endogenous IAA, the content of bioactive GAs, and the level of ABA.

### Sufficient energy supply is key to microspore development

The energy supply is crucial for microspore development. Mitochondria are important sites for energy production [53]. The electron transport chain in the mitochondrial membrane is the main site of ROS production [54]. ROS are involved in the regulation of reproductive growth in plants. During microspore development, abnormal ROS accumulation leads to programmed cell death. H_2_O_2_, MDA, O_2_·^**−**^, CAT, and POD are important indicators of the response to ROS levels. H_2_O_2_ stimulates the formation of embryonic cells [55]. Several studies have shown that H_2_O_2_ accumulation can produce a stress response, which has a promotional effect on embryonic development. The level of antioxidant enzyme activity plays an important role in scavenging free radicals and regulating cellular tissue activity during embryogenesis[26]. In this study, the results showed that the contents of MDA, O_2_·^**−**^, and H_2_O_2_ were lower than those of the control group, and the activities of CAT and POD were higher than those of the control group, which were consistent with the results of previous studies on crops such as *platycodon grandiflorum* [56, 57]. This indicated that production and removal of reactive oxygen species in the bud were balanced and the damage caused by membrane lipid peroxidation was prevented.

NADH dehydrogenase and succinate dehydrogenase (SDH) play important roles in mitochondrial electron transport. NADH dehydrogenase complex 1 was an enzyme that catalyzes the transfer of electrons from NADH to CoQ at the inner mitochondrial membrane, and was the “entry enzyme” for mitochondrial oxidative phosphorylation [58]. Succinate dehydrogenase (SDH) was one of the hubs that connects electron transport and oxidative phosphorylation, providing electrons for the respiratory chain [59]. Higher plant cells had two F1-F0 ATPase complexes that were involved in mitochondrial oxidative phosphorylation. The F1F0-ATP complex enzyme can catalyze the hydrolysis or synthesis of ATP by adjusting the electrochemical potential gradient through proton penetration [60]. The accumulation of cytoplasmic PPI was toxic and can lead to serious growth defects and even cell death [61]. PPAs were enzymes that mainly participate in the hydrolysis of PPI into two inorganic phosphates (PI), which keep plant cells at a low level of cytoplasmic PPI [62]. In this study, most of the DEGs related to electron transport in the oxidative phosphorylation pathway were upregulated genes, including *SDH*, *SAH*, *PPA*, *AHA*, and *VHA*. NADH and succinate are dehydrogenated by NADH dehydrogenase and succinate dehydrogenase, respectively, which release protons and accelerate the efficiency of the electron transport chain, respectively, Catalyzed by PPA enzymes, PPi was hydrolyzed to Pi and ADP, while the upregulated expression of *VHA* and *AHA* accelerated the rate of electron transfer inside and outside the mitochondria and promoted the ATP synthesis [63]. We speculated that the upregulation of *SDH*, *SAH*, *PPA*, *AHA*, and *VHA* accelerated the electron transport rate inside and outside of the mitochondria, inhibited the generation of ROS, improved the efficiency of mitochondrial oxidative phosphorylation and the production efficiency of ATP, thus providing sufficient energy for the development of microspores, which might be the important reason for the accelerated rate of isolated microspore embryogenesis. It should be noted that carbohydrates such as sugars, as energy donors, played an important role in the growth and development of plants. In this study, after treatment with 100 mg. L^− 1^ IAA, the contents of soluble total sugar, amino acids, starch and soluble protein in flower buds at the late uni-nucleus stage were markedly higher than those in the control group. This might further explain that the adequate supply of energy was the important reason for the increase of the incidence of isolated microspore embryogenesis.

### Degradation of the cell wall may have accelerated microspore development

Cell proliferation, expansion, and differentiation are the essential cytological basis for plant growth and development. There is a close link between this series of cytological events and cell wall metabolism. As a significant component of the primary cell wall of higher plants, the metabolism of pectin modification, synthesis, and degradation was involved in numerous aspects of plant growth and development by affecting the mobility and malleability of the cell wall [63]. Pectin methyl esterase (*PME*) demethylates pectin to form pectic acid and methanol, which alters the pH value of the cell wall and relax it [64]. Several *PME* genes are involved in pollen development in Arabidopsis and cabbage[65]. Polygalacturonase (*PGs*) uses polygalacturonic acid as the substrate. It acts on alpha-1, 4-D- galactosides bond to hydrolyze the pectic acid on the polygalacturonic acid main chain into galacturonic acid, galacturonic acid, the main component of pectin in the plant cell wall, is degraded into a single polygalactose residue which exists in tapetum, sporophyte, and pollen [66, 67]. In this study, almost all *PME* and *PGs* were upregulated, and in addition, the pectin content was significantly lower than that in the control group. Therefore, we speculated that upregulation of key genes involved in pectinase accelerated pectin degradation, resulting in a decrease in pectin content, degradation of the cell wall of pollen mother cells, and accelerated release of tetrad microspores. Pectate lyase (*PLY*) is present in anthers and pollen and can loosen cell walls [68]. USP catalyzes the reversible conversion of D-glucuronate-1P to UDP-D-glucuronate and ultimately UDP-glucose synthesis. Studies have shown that a deficiency in USP activity leads to male sterility [69–71]. USP is involved in the rapid growth of plant cells [72].

In this study, 75% of *PLY* and *USP* were upregulated genes, and we speculate that upregulation of *PLY* may lead to partial autolysis of cell wall and loosening of cell wall. The upregulation of *USP* may have accelerated cell growth, which may further lead to an increased incidence of isolated microspore embryogenesis.

### The significant expression of DEGs may be related to microspore embryogenesis

Exploring the genes involved in the initiation of microspore embryos will help to better understand the molecular mechanism of the molecular mechanism of microspore embryogenesis. In this study, we further screened 139 DEGs from genes related to metabolic pathways and development, 97 of which were upregulated. *WUS*, *GSTU12*, *PEX3*, *PRK3, BGL13*, *BFRUCT3*, *PME5*, *CYP707A3*, *AHA8*, *AHA9*, *PPA3*, and *PPA6* were highly expressed in the IAA treated group. *WUS* plays an important role in the embryonic development of *Arabidopsis thaliana* [73], and a study has shown that *GST* is related to the embryonic development of *sorghum* [74]. We speculated that these genes were related to the occurrence and development of microspore embryos, and further research was needed.

## Conclusions

In this study, we first determined that 100 mg·L^− 1^ IAA was the optimal hormone concentration for inducing isolated microspore embryogenesis by counting the embryo yield. Cytological observation and the statistics of initial time of embryogenesis indicated that IAA treatment accelerated the development of microspores. Treatment with exogenous IAA caused changes in the content and dynamic balance of endogenous plant hormones. We have focused on the transcript and pathway, particularly affecting embryonic development. Transcriptome analysis, it was found that most genes related to GA and Auxin (IAA) synthesis and signal transduction, *PMEs* and *PGs* genes, and genes related to ATP synthesis and electron transport chain were upregulated, and genes related to ABA synthesis and signal transduction were downregulated. Physiological and biochemical indexes showed that exogenous IAA altered the contents of soluble total sugars, amino acids, starch, soluble protein, malondialdehyde and pectin, CAT and POD activities, and the production rates of H_2_O_2_ and O2·^−^. These results indicated that exogenous IAA treatment could change the balance of endogenous hormones, accelerate cell wall degradation, promote ATP synthesis and nutrient accumulation, and inhibit ROS accumulation, which ultimately promotes microspore embryogenesis.

## Materials and methods

### Plant material and microspore culture

Line Huiwu (HW) was screened by Chenggang Wang, and all seed materials were kept in the Vegetable Breeding Laboratory of the College of Horticulture, Anhui Agricultural University (Hefei, Anhui Province, China). No specific permits were required for the described field studies. The location is not privately-owned or protected in any way, and the field studies did not involve endangered or protected species.

The Wucai varieties HW grew under 25℃ and light intensity was 20,000 lx conditions in the plastic greenhouse of the Wanjiang Vegetable Industry and Technology Research Institute (Ma’anshan, Anhui Province, China) in the spring.

At the full bloom stage (25-75% of the whole plant flowers open period), the buds at the mononuclear stage were treated with 0 mg·L^− 1^, 50 mg·L^− 1^, 75 mg·L^− 1^, 100 mg·L^− 1^, 125 mg·L^− 1^, 150 mg·L^− 1^ IAA and collected after 24 h. Bud pre-treatment was performed according to method of Zhang et al. [75]. In short, the collected buds were first sterilized with 75% alcohol for 30 s, then with 0.1% mercuric chloride for 6 min, and finally rinsed with sterile fresh water three times for 5 min each time. Microspores were isolated from the anthers by grinding the buds in B_5_ liquid medium using sterile glass rods and filtering the suspension through a 30 μm nylon mesh. Centrifugation was performed three times (800×g, 3 min each), and the microspores were resuspended in isolation medium. The centrifuged microspores were collected and resuspended in NLN-13 medium, with 13% sucrose and buffered at pH 5.8.The microspore density was adjusted to 1 × 10^5^ microspores per milliliter using a hemocytometer (AP-0650010,MARIENFELD, Germany) [76]. The microspore suspension was dispensed into 60 mm × 15 mm Petri dishes with 5 mL per plate, incubated in the dark at 33℃ for 24 h and then transferred to the dark at 25 ℃ for embryonic development.

### Cytological observation of microspore development

Cytological examination of the microspores for the first two weeks of culture after treatment with 100 mg·L^− 1^ IAA and the control group. Representative microspores cultured for 0,3,5,10, and 15 days were selected from the IAA treatment and the control group respectively, with three replicates. The microspores in 3 petri dishes were randomly selected together with the medium and inhaled into a 20 mL centrifuge tube, centrifuged at 800 rpm for 4 min, and the supernatant was discarded. After collection, the microspores were sucked onto the slide, covered with the cover slide, cut into pieces, and observed under an inverted microscope.

### Determination of endogenous plant hormone content

Six hormones were selected and determined in flower buds treated with 100 mg. L^− 1^ IAA or the fresh water respectively by using LC-MS/MS method, including Auxin, CKs, GA, JA and ABA. 0.3 g fresh buds were collected, immediately frozen in liquid nitrogen, ground into powder (30 Hz, 1 min), and stored at -80 °C. 50 mg of plant sample was weighed into a 2 mL plastic microtube and frozen in liquid nitrogen, dissolved in 1 mL methanol/water/formic acid (15:4:1, V/V/V). 10 µL internal standard mixed solution (100 ng/mL) was added into the extract as internal standards (IS) for the quantication. The mixture was vortexed for 10 min, then centrifugation for 5 min (12,000 r/min, and 4 °C), the supernatant was transferred to clean plastic microtubes, followed by evaporation to dryness and dissolved in 100 µL 80% methanol (V/V), and filtered through a 0.22 μm membrane filter for further LC-MS/MS analysis [77–79].

The chromatographic column was a Waters ACQUITY UPLC HSS T3 C18, 1.8 μm, 2.1 mm × 100 mm. The mobile phase (phase A) was ultrapure water with 0.04% acetic acid, and the organic phase (phase B) was acetonitrile with 0.04% acetic acid. The elution gradient was set as follows: 0 min, A: B = 95:5 (V: V); 1 min, A: B = 95:5 (V: V); 8 min, 5:95 (V: V); 9 min, 5:95 (V: V); 9.1 min, 95:5 (V: V); and 12 min, 95:5 (V: V). The flow rate was 0.35 mL/min, the column temperature was 40 ◦C, and the injection volume was 2 µL.

Mass detection using Q-Trap 6500+ (SCIEX, USA). The mass spectrometry conditions were as follows: electrospray ionization (ESI) temperature, 550℃; mass spectrum voltage, 5500 V; curtain gas (CUR), 35 psi. Linear ion trap (LIT) and triple quadrupole (QQQ) scans were acquired on a triple quadrupole-linear ion trap mass spectrometer (QTRAP), QTRAP® 6500 + LC-MS/MS System, equipped with an ESI Turbo Ion-Spray interface, operating in both positive and negative ion mode and controlled by Analyst 1.6.3 software (Sciex). Multiquant 3.0.3 software (Sciex) was used to quantify all metabolites [80–82].

### Transcriptome analysis and gene annotation

Total RNA from buds of treatment with 100 mg·L^− 1^ IAA and the control group were extracted using RNAprep Pure Plant Plus kit (Tiangen Biotech, Beijing, China) according to the manufacturer’s instructions. RNA degradation and contamination was monitored on 1% agarose, RNA purity was checked using the NanoPhotometer® spectrophotometer (IMPLEN, CA, USA). RNA concentration was measured using Qubit® RNA Assay Kit in Qubit®2.0 Flurometer (Life Technologies, CA, USA). RNA integrity was assessed using the RNA Nano 6000 Assay Kit of the Bioanalyzer 2100 system (Agilent Technologies, CA. USA). Sequencing libraries were generated using NEBNext® UltraTM RNA Library Prep Kit for Illumina® (NEB, USA) following manufacturer’s recommendations and index codes were added to attribute sequences to each sample. After the completion of library construction, the Qubit2.0 Fluorometer was used for initial quantification, followed by Agilent 2100 bioanalyzer for insert size of the library. After the insert size met the expectation, qRT-PCR accurately quantified the effective concentration of the library (the effective concentration of the library was higher than 2nM) to ensure the quality of the library. The clustering of the index-coded samples was performed on a Bot Cluster Generation System using TruSeq PE Cluster Kit v3-cBot-HS (Illumia) according to the manufacturer’s instructions. After cluster generation, the library preparations were sequenced on an Illumina platform and 150 bp paired-end. Use fastp v 0.19.3 to filter the original data, mainly to remove reads with adapters, all subsequent analyses are based on clean reads. Use HISAT v2.1.0 to compare clean reads with *Brassica rapa* v 3.0 reference genome. Use String Tie v1.3.4d for new gene (genes not compared to reference genome) prediction. Use featureCounts v1.6.2 /StringTie v1.3.4d to calculate the gene alignment and FPKM. DESeq2 v1.22.1 /edgeR v3.24.3 was used to analyze the differential expression between the two groups, and the *P-value* was corrected using the Benjamini & Hochberg method. The corrected *P-value* and |log_2_foldchange| are used as the threshold for significant difference expression. The enrichment analysis was performed based on the hypergeometric test. For KEGG, the hypergeometric distribution test is performed with the unit of pathway; for GO, it was performed based on the GO term.

### Bud physiological indicators and enzyme activity determination

The buds were treated with 100 mg. L^− 1^ of IAA between 8: 00 a.m. and 9: 00 a.m. at the full bloom stage (25-75% flowering period of the whole plant). The control treatment was sprayed with fresh water under the same conditions. After 24 h, buds with the largest population of late-uninucleate-stage microspores stage were selected for determination of soluble sugar, soluble protein, starch, ROS related enzymes and Oxidative damage index. The experiment was repeated three times. The soluble sugar content was determined by an anthrone colorimetric method, soluble protein content was determined by Coomassie brilliant blue G-250 method [83], and starch content was determined by perchloric acid extraction-continuous flow method [84].

A centrifuge tube was used to collect 50 mg dry flower bud sample, which was mixed with 4 mL of 80% ethanol. The sample was treated with a fresh water bath at 80 ℃ for 30 min, cooled and centrifuged at 5000×g for 3 min. The supernatant was collected, and the residue was washed with 80% ethanol three times. After each washing, the supernatants were extracted and combined. Then 10 mg of activated carbon was added, decolorized at 80 ℃ for 30 min, diluted to 10 mL, and then filtered to obtain the extract.

#### Determination of soluble total sugar

20 µL of the above extractive solution was combined with 480 µL of fresh water and 2.5 mL of anthrone, mixed, reacted in a 90℃ fresh water bath for 15 min, taken out, cooled rapidly, and develop for 10 min before being measured at the wavelength of 620 nm.

#### Determination of starch content

10 mL of 52% perchloric acid was added into the above residue, the residue was centrifuged at 4000×g for 3 min, and the supernatant was collected. Then 10 mg of activated carbon was added, and the residue was treated with a fresh water bath at 80℃ for 30 min. After decolorization, filtration was performed, 5 mL of anthrone was added into 2 mL of filtrate, and the filtrate was shaken up immediately. Then the filtrate was treated with a fresh water bath at 80℃ for 10 min, cooled, developed in the dark for 10 min, and then the absorbance value was measured at a wavelength of 620 nm.

#### Determination of soluble protein

After washing, 0.2 g of fresh buds were placed in a pre-cooled mortar, 1.6 mL of 5 mmol. L^− 1^ pre-cooled phosphate buffer solution (pH 7.8) was added and ground on an ice bath to homogenate, then transferred to a centrifuge tube for centrifugation at 4℃ and 12,000×g for 20 min. To 20 uL of the extract, 80 uL of fresh water was added, followed by 2.9 mL of Coomassie brilliant blue solution. After reacting for 2 min, the absorbance was measured at 595 nm.

### Determination of amino acid, CAT, POD, MDA, O_2_·^−^, H_2_O_2_, and Protopectin content

Amino acid (AA), MDA content, superoxide anion (O_2_·^**−**^), hydrogen peroxide (H_2_O_2_) content and catalase (CAT), peroxidase (POD) activities, Protopectin content were determined using Solarbio reagent kits (BC1570, BC0020, BC0290, BC3595, BC0205, BC0090, BC3685; Solarbio, Beijing, China). Each sample (fresh buds) about 50–200 mg (fresh weight) thoroughly ground for the determination of the above indicators.

### RT-qPCR identification

RNA was isolated from the flower buds with single nucleus sidelined treated with 100 mg. L^− 1^ IAA and treated with the same dose of fresh water, and DNase-treated RNA (1 mg) was reverse transcribed into cDNA using a PrimeScript™ RT kit (TaKaRa, Japan). RT-qPCR was performed using SYBR® Premix Ex Taq™ II kit (TaKaRa, Japan). PCR amplification was performed in a Bio-Rad CFX96 instrument according to the manufacturer’s instructions. Three biological replicates were performed for each sample, and three technical replicates were performed for each gene. The data were standardized with the expression level of *BnaActin* as the control. The relative expression levels were calculated using the 2 ^−ΔΔCt^ method. Primer Software Version v6.0 (Premier Biosoft International, Palo Alto, CA, USA) was used to design primers, and primer sequences were listed in additional file 8: Table s4.

### Data analysis

All data are expressed as the mean ± standard deviation, using at least three biological replicates. Data were statistically analyzed using spass.26 (SPSS Institute, Chicago, IL, USA) software, and means were compared using the *t* tests with a significance level of 0.05. Graphs were drawn using GraphPad Prism software (GraphPad Software Inc., USA), Origin 2018 64 Bit, and Adobe Illustrator CC 2019.

## Electronic supplementary material

Below is the link to the electronic supplementary material.

Supplementary Material 1

Supplementary Material 2

Supplementary Material 3

Supplementary Material 4

Supplementary Material 5

Supplementary Material 6

Supplementary Material 7

Supplementary Material 8

## Data Availability

The raw RNA-Seq data used in this study have been deposited in the Nation Center for Biotechnology Information (NCBI) Sequence Read Archive (SRA) database under the accession number PRJNA933255 (https://www.ncbi.nlm.nih.gov/sra/ PRJNA933255).

## Abbreviations

IAA: Indoleacetic acid
DEGs: Differentially Expressed Genes
KEGG: Kyoto Encyclopedia of Genes
GO: Gene Ontology
qRT-PCR: Quantitative real-time polymerase chain reaction
RNA-Seq: RNA sequencing technology
FPKM: The expected number of fragments per kilobase of transcripts per million mapped fragments
DHs: Doubled haploids
WUS: WUECHEL
LTP: non-specific lipid transferase protein
GST: glutathione S-transferase
GA: gibberellin
CK: Cytokinin
ABA: Abscisic acid
PCD: Programmed cell death
ROS: Reactive Oxygen Species
JAs: Jasmonic acid
SA: Salicylic acid
SLs: Strigolactones
RALF: protein RALF-like
IAM: indole 3 acetamide
IAAld: indole 3 acetaldehyde
ALDH: aldehyde dehydrogenase
LUT5: β-cyclohydroxylase
DWARF27: β-carotene isomerase
AOG: abscisic β-glucosyltransferase
CYP707A: abscisic acid 8’-hydroxylase
PYL: abscisic acid receptor
PP2C: protein phosphatase
SDH: succinic dehydrogenase
VHA: V-type proton ATPase
PPA: pyrophosphate
USP: UDP- sugar pyrophosphorylase
NCED: 9-Cis-epoxycarotenoid dioxygenase
Pi: inorganic phosphates
PME: Pectin methyl esterase
PGs: Polygalacturonase
PLY: Pectate lyase
AA: Amino acid
CAT: Catalase
POD: Peroxidase
H_2_O_2_: hydrogen peroxide
