# Supplementary material for: Exogenous spraying of IAA improved the efficiency of microspore embryogenesis in Wucai (Brassica campestris L.) by affecting the balance of endogenous hormones, energy metabolism, and cell wall degradation
Source: BMC Genomics. 2023 Jul 6;24:380. doi: 10.1186/s12864-023-09483-2 (PMC10327361; doi:10.1186/s12864-023-09483-2)
Supplement: Supplementary file 1 — Supplementary Material 1 [file 12864_2023_9483_MOESM1_ESM.docx]

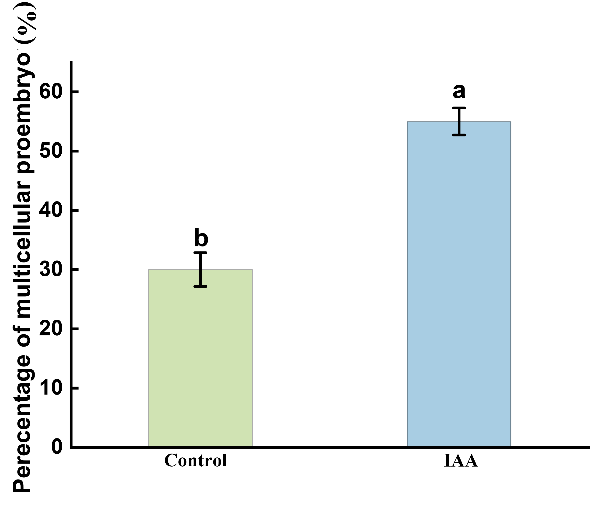


Fig s1. Percentage of multicellular proembryo to total cell culture

Note: Error bars represent± SD. Different letters indicate significant differences (*p* < 0.05).
