# Supplementary material for: Exogenous spraying of IAA improved the efficiency of microspore embryogenesis in Wucai (Brassica campestris L.) by affecting the balance of endogenous hormones, energy metabolism, and cell wall degradation
Source: BMC Genomics. 2023 Jul 6;24:380. doi: 10.1186/s12864-023-09483-2 (PMC10327361; doi:10.1186/s12864-023-09483-2)
Supplement: Supplementary file 2 — Supplementary Material 2 [file 12864_2023_9483_MOESM2_ESM.docx]

Table s1 Statistics of initial time of microspore embryogenesis

| IAA concentration (mg^.^ L^-1^) | Start date of microspore culture | Start date of microspore embryogenesis |
| --- | --- | --- |
| 0 | March 2nd | March 18 |
| 50 | March 2nd | March 17 |
| 75 | March 2nd | March 16 |
| 100 | March 2nd | March 14 |
| 125 | March 2nd | March 16 |
| 150 | March 2nd | March 16 |
