# Supplementary material for: Exogenous spraying of IAA improved the efficiency of microspore embryogenesis in Wucai (Brassica campestris L.) by affecting the balance of endogenous hormones, energy metabolism, and cell wall degradation
Source: BMC Genomics. 2023 Jul 6;24:380. doi: 10.1186/s12864-023-09483-2 (PMC10327361; doi:10.1186/s12864-023-09483-2)
Supplement: Supplementary file 3 — Supplementary Material 3 [file 12864_2023_9483_MOESM3_ESM.docx]

Table s2. Ratio of different endogenous plant hormone

| Hormone ratio | Control (ng/g) | IAA (ng/g) |
| --- | --- | --- |
| (Auxin+GAs)/ABA | 88.79 ± 13.8 b | 248.779 ± 11.9 a |
| (Auxin+GAs)/CK | 235.26 ± 8.4 a | 240.3 ± 12.4 a |
| (Auxin+GAs+CK)/ABA | 89.16 ± 13.2 b | 249.82 ± 11.9 a |
| Auxin/CK | 233.59 ± 8.2 a | 237.41 ± 12.19 a |
| Auxin/GAs | 143.00 ± 12.1 a | 81.84 ± 1.97 b |
| Auxin/ABA | 88.14 ± 13.6 b | 245.78 ± 21.7 a |
| CK/ABA | 0.37 ± 0.04 b | 1.04 ± 0.1 a |
| GAs/ABA | 0.65 ± 0.2 b | 3.01 ± 0.2 a |
| CK/GAs | 0.62 ± 0.1 a | 0.35 ± 0.03 b |

Note: means followed by different letters are significantly different at *p*<0.05 level
