# Supplementary material for: Exogenous spraying of IAA improved the efficiency of microspore embryogenesis in Wucai (Brassica campestris L.) by affecting the balance of endogenous hormones, energy metabolism, and cell wall degradation
Source: BMC Genomics. 2023 Jul 6;24:380. doi: 10.1186/s12864-023-09483-2 (PMC10327361; doi:10.1186/s12864-023-09483-2)
Supplement: Supplementary file 4 — Supplementary Material 4 [file 12864_2023_9483_MOESM4_ESM.docx]

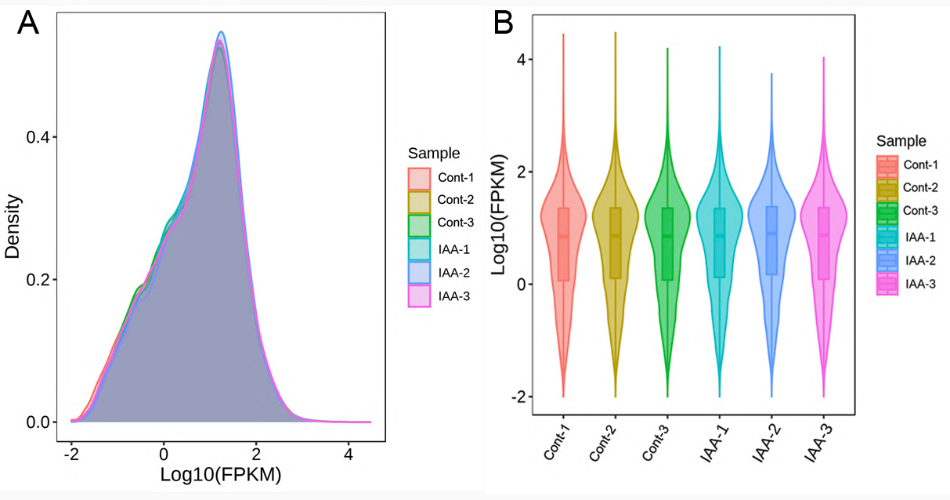


Fig. s2 The FPKM density distribution curve and box diagram of each sample. (a) Different colored curves in the figure represent different samples. The abscissa of each point on the curve represents the logarithm of the corresponding sample FPKM, and the ordinate of each point represents the probability density. (b) The different colors in the figures represent different samples. The abscissa is the name of the sample, and the ordinate is log10 (FPKM). The width of each violin figure reflects the number of genes at this expression level.
