# Supplementary figures and images for: Exogenous spraying of IAA improved the efficiency of microspore embryogenesis in Wucai (Brassica campestris L.) by affecting the balance of endogenous hormones, energy metabolism, and cell wall degradation
Source: BMC Genomics. 2023 Jul 6;24:380. doi: 10.1186/s12864-023-09483-2 (PMC10327361; doi:10.1186/s12864-023-09483-2)

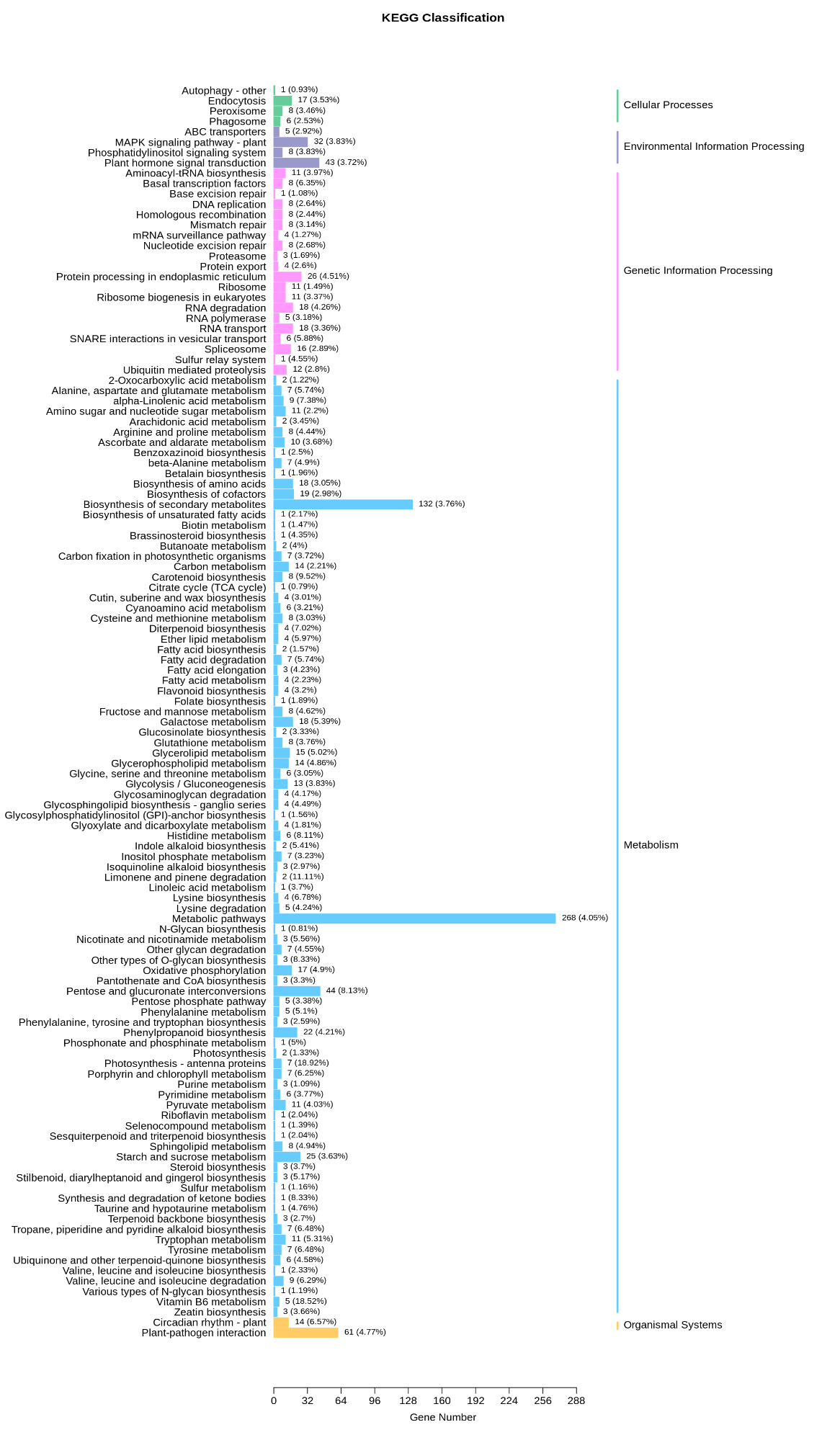


Fig s3 KEGG enrichment map for DEGs

Supplement: Supplementary file 5 — Supplementary Material 5 [file 12864_2023_9483_MOESM5_ESM.docx]
