# Supplementary material for: Exogenous spraying of IAA improved the efficiency of microspore embryogenesis in Wucai (Brassica campestris L.) by affecting the balance of endogenous hormones, energy metabolism, and cell wall degradation
Source: BMC Genomics. 2023 Jul 6;24:380. doi: 10.1186/s12864-023-09483-2 (PMC10327361; doi:10.1186/s12864-023-09483-2)
Supplement: Supplementary file 6 — Supplementary Material 6 [file 12864_2023_9483_MOESM6_ESM.docx]

Table s3 Identification of DEGs associated with anther, pollen and embryo development in Wucai

| Brassica Gene | log2FC | up/down | Gene Name | Gene description |
| --- | --- | --- | --- | --- |
| BraA07g029130.3C | 3.99921414 | up | PRK3 | pollen receptor-like kinase 3 |
| BraA08g013020.3C | 3.11393527 | up | OLE96 | pollen-specific protein-like At4g18596 |
| BraA03g042940.3C | 3.05050855 | up | PLRX3 | pollen-specific leucine-rich repeat extensin-like protein 3 |
| BraA09g035740.3C | 2.46187431 | up | OLE96 | pollen-specific protein-like At4g18596 |
| BraA02g032230.3C | 2.41011890 | up | OLE96 | pollen-specific protein-like At4g18596 |
| novel.810 | 2.11258037 | up | GFT1 | pollen coat protein class B 1 |
| BraA06g042170.3C | 2.07826406 | up | OLE96 | pollen-specific protein-like At4g18596 |
| novel.2905 | 2.04538645 | up | -- | pollen coat protein [Brassica oleracea var. alboglabra] |
| novel.3487 | 1.89434184 | up | -- | pollen coat protein class A no. 1 |
| BraA09g003030.3C | 1.67056278 | up | -- | pollen allergen Che a 1-like |
| BraA08g024080.3C | 1.59348244 | up | OLE96 | pollen-specific protein-like At4g18596 |
| BraA01g010110.3C | 1.17849241 | up | OLE96 | pollen-specific protein-like At4g18596 |
| BraA06g037940.3C | 1.10845413 | up | -- | pollen-specific protein-like At4g18596 |
| BraA06g011490.3C | 2.22155571 | up | LEA1 | late embryogenesis abundant protein 1 |
| BraA08g002000.3C | 1.63505086 | up | LEA1 | late embryogenesis abundant protein 1-like |
| BraA06g001880.3C | 1.20461302 | up | LEA | late embryogenesis abundant protein D-7 |
| BraA01g035360.3C | 3.27462731 | up | PMEI2 | pectinesterase inhibitor 2-like |
| BraA05g000800.3C | 2.73630437 | up | PME5 | pectinesterase 5 |
| BraA04g032380.3C | 2.17049849 | up | PME5 | pectinesterase 5-like |
| BraA05g000810.3C | 2.12619660 | up | PME4 | pectinesterase 5-like |
| BraA10g029820.3C | 2.09774029 | up | PPME1 | pectinesterase PPME1 |
| BraA04g032370.3C | 2.05085918 | up | PME5 | pectinesterase 5-like |
| BraA09g051660.3C | 1.83468074 | up | PME | Probable pectinesterase inhibitor |
| BraA03g024320.3C | 1.78474612 | up | PME4 | pectinesterase 5 |
| BraA04g000830.3C | 1.76265888 | up | PME37 | probable pectinesterase inhibitor VGDH2 isoform X1 |
| BraA10g029810.3C | 1.68607823 | up | PME49 | probable pectinesterase 49 |
| BraA03g025430.3C | 1.68321675 | up | PME13 | probable pectinesterase inhibitor 13 |
| BraA02g040020.3C | 1.67001024 | up | PME58 | probable pectinesterase inhibitor 58 |
| BraA03g032200.3C | 1.57622611 | up | PME21 | probable pectinesterase inhibitor 21 |
| BraA09g051670.3C | 1.53284635 | up | PME | probable pectinesterase inhibitor |
| BraA09g001840.3C | 1.26929758 | up | PME41 | probable pectinesterase inhibitor 41 |
| BraA03g036380.3C | 1.13442727 | up | PME3 | pectinesterase inhibitor 3-like |
| BraA06g024960.3C | 1.06698532 | up | PME34 | probable pectinesterase inhibitor 34 |
| BraA10g021360.3C | 3.63979284 | up | EDL16 | sugar transporter ERD6-like 16 |
| BraA03g027420.3C | 1.79794567 | up | SWET7 | bidirectional sugar transporter SWEET7-like |
| BraA05g002110.3C | 2.90175894 | up | BH100 | transcription factor bHLH100-like |
| BraA05g039550.3C | 1.48663531 | up | BH150 | transcription factor bHLH150-like |
| BraA03g038020.3C | 1.71206029 | up | CHX19 | cation/H(+) antiporter 19-like |
| BraA09g003410.3C | 2.14057615 | up | GATLA | probable galacturonosyltransferase-like 10 |
| BraA06g037150.3C | 2.03105174 | up | GATLA | probable galacturonosyltransferase-like 10 |
| novel.3050 | 2.79383719 | up | RLF9 | protein RALF-like 9 |
| BraA01g029230.3C | 2.14326454 | up | RLF15 | protein RALF-like 15 |
| novel.223 | 2.09064288 | up | RLF9 | protein RALF-like 9 |
| BraA07g011780.3C | 1.93963961 | up | RLF4 | protein RALF-like 4 |
| BraA08g024410.3C | 1.85517632 | up | RLF4 | protein RALF-like 4 |
| novel.1775 | 1.82512005 | up | RALF13 | protein RALF-like 13 |
| BraA09g036450.3C | 1.73282942 | up | RLF4 | protein RALF-like 4 |
| BraA03g017550.3C | 1.55190289 | up | RLF17 | protein RALF-like 17 |
| BraA07g007880.3C | 1.27882669 | up | RLF26 | protein RALF-like 26 |
| BraA07g011590.3C | 3.39653364 | up | KTI2 | cysteine protease inhibitor WSCP-like |
| BraA07g034810.3C | 2.49229563 | up | GSTUC | glutathione S-transferase U12 |
| BraA03g016430.3C | 1.12367852 | up | GSTFA | glutathione S-transferase F10 |
| BraA07g002990.3C | 3.03270203 | up | WUS | protein WUSCHEL-like |
| BraA03g038230.3C | 1.66455804 | up | WOX1 | WUSCHEL-related homeobox 1-like isoform X2 |
| BraA09g011150.3C | 1.45031818 | up | WUS | protein WUSCHEL-like |
| BraA06g016850.3C | 2.01872876 | up | GLTP2 | glycolipid transfer protein 2 |
| BraA09g038140.3C | -1.0206890 | down | RAVL1 | AP2/ERF and B3 domain-containing transcription repressor TEM1-like |
| BraA09g060240.3C | -1.0860413 | down | RAV1 | AP2/ERF and B3 domain-containing transcription factor RAV1-like |
| BraA03g060570.3C | -1.3045153 | down | RA210 | ethylene-responsive transcription factor RAP2-10-like |
| BraA08g025570.3C | -1.6214858 | down | RAVL1 | AP2/ERF and B3 domain-containing transcription repressor TEM1-like |
| BraA05g024730.3C | -8.4939650 | down | LEA31 | late embryogenesis abundant protein 31 |
| BraA06g001110.3C | -1.3918702 | down | PME1 | pectinesterase 1 |
| BraA04g012750.3C | -1.1860623 | down | SWET8 | bidirectional sugar transporter SWEET8 |
| BraA02g043840.3C | -1.9716108 | down | SWET5 | bidirectional sugar transporter SWEET5-like |
| BraA08g011740.3C | -1.0069947 | down | BH147 | transcription factor bHLH147-like |
| BraA01g006790.3C | -1.0861500 | down | BHLH69 | transcription factor bHLH69 |
| BraA06g006160.3C | -1.3318528 | down | BH149 | transcription factor bHLH149 |
| BraA06g041630.3C | -1.5398542 | down | BH028 | transcription factor bHLH28-like |
| BraA08g026800.3C | -1.7982759 | down | BH094 | transcription factor bHLH94-like |
| BraA03g059850.3C | -1.8016760 | down | BH011 | transcription factor bHLH11 |
| BraA02g012460.3C | -2.11195499 | down | BH035 | transcription factor bHLH35-like |
| BraA03g013630.3C | -1.1800064 | down | GDL87 | GDSL esterase/lipase At5g55050-like |
| BraA07g029660.3C | -2.4237074 | down | GDL28 | GDSL esterase/lipase At1g71250-like |
| BraA10g013070.3C | -2.7541580 | down | GDL87 | GDSL esterase/lipase At5g55050 |
| BraA03g040910.3C | -1.3163163 | down | NIPA1 | probable magnesium transporter NIPA1 |
| BraA06g012990.3C | -1.4745503 | down | GSTUQ | glutathione S-transferase U26 |
| BraA03g024700.3C | -2.1478284 | down | GSTU5 | glutathione S-transferase U5 |
| BraA05g014700.3C | -2.9105026 | down | GSTU5 | glutathione S-transferase U5-like |
| novel.2616 | -8.6079276 | down | GSTT3 | glutathione S-transferase T3-like |
