# Supplementary material for: Exogenous spraying of IAA improved the efficiency of microspore embryogenesis in Wucai (Brassica campestris L.) by affecting the balance of endogenous hormones, energy metabolism, and cell wall degradation
Source: BMC Genomics. 2023 Jul 6;24:380. doi: 10.1186/s12864-023-09483-2 (PMC10327361; doi:10.1186/s12864-023-09483-2)
Supplement: Supplementary file 7 — Supplementary Material 7 [file 12864_2023_9483_MOESM7_ESM.docx]

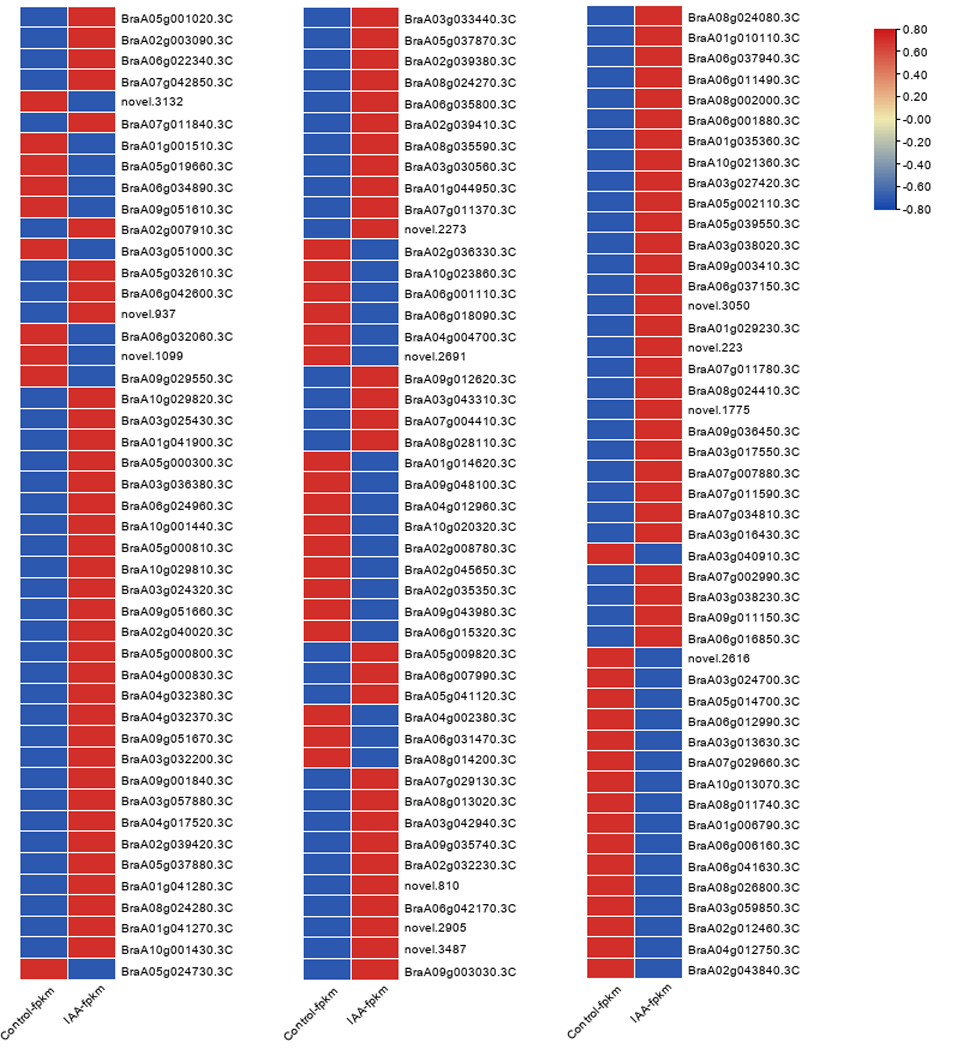


Fig s4. Heat map of DEGs.

Note: Each row represents a gene, red represents upregulation, and blue represents downregulation.
