# Supplementary material for: Exogenous spraying of IAA improved the efficiency of microspore embryogenesis in Wucai (Brassica campestris L.) by affecting the balance of endogenous hormones, energy metabolism, and cell wall degradation
Source: BMC Genomics. 2023 Jul 6;24:380. doi: 10.1186/s12864-023-09483-2 (PMC10327361; doi:10.1186/s12864-023-09483-2)
Supplement: Supplementary file 8 — Supplementary Material 8 [file 12864_2023_9483_MOESM8_ESM.docx]

Table S4 Primers used in this study

| Gene name | Primer name | Primer sequences (5’-3’) |
| --- | --- | --- |
| BraA05g000800.3C (PME5) | F | TCATTCTATGTATCGCCC |
|  | R | TTGTTCAGCTGTTTCTCG |
| BraA09g013410.3C (D27) | F | AGAACGAAATAAAGGAGA |
|  | R | AGAAAGGCACTGTAAGAG |
| BraA01g035920.3C (IAA26) | F | TAAACGAATCCTCCCACG |
|  | R | TTGCTTCACACCATCACC |
| BraA07g020250.3C (SDHB2) | F | CGAAAGAGACGACGATCACG |
|  | R | AATCTCCTTCCCAGGAACCG |
| BraA06g010270.3C (DAO) | F | AACAGTTGGTAAAAATGG |
|  | R | GAAGGTGGAGATGGTAAT |
| BraA08g023600.3C (GA2OX2) | F | TAATCCCAACATACAAAC |
|  | R | AACCAAACTCTTCACAGG |
| BraA06g032060.3C (CYP71AB) | F | ATGCTCCTTCACTTTGGT |
|  | R | GCTATTTCTGTCCCTTTA |
| BraA08g035590.3C (PGLR1) | F | GCACAATCACCGCTACAC |
|  | R | TCCATACGGCTTCACCTT |
| BraA03g030560.3C (PLY7) | F | TGTGTGTGATTTGTATTG |
|  | R | GCCTCCTGTGGTCCTGTA |
| BraA06g042600.3C (ABAH3) | F | TCTCTGTTTGCTCCGGCTAA |
|  | R | GAGTCTTGAACACCGAACCG |
| BraA08g027750.3C (CATA3) | F | GCGCCGAGAAAGTTCCTATC |
|  | R | AGACGTGGTTCCGACAGAAT |
| BraA02g017430.3C (C7352) | F | CGTGAGGTCCGACAAGTTTC |
|  | R | AGTGGCAGGAGGGTAAAGTC |
| BraA09g051660.3C (BP19) | F | GTGGCGACAAGACTCTGAAC |
|  | R | TGAAGCCGTGAAGTTTGTGG |
| BraA01g001510.3C (GH3.3) | F | TCAATCTCGGACGACCTCTG |
|  | R | ACGGGAACCTTGGTCTTGAA |
| BraA07g042850.3C (PMA9) | F | AGTCTCCATCACCATCCG |
|  | R | CAACAGTCATCACCGCAA |
| BraA06g001110.3C (PME1) | F | CTAGACGAGCTGGCTCAGAA |
|  | R | GAACTCCGAAGTCGCTCAAC |
